# Effect of imbalanced sampling and missing data on associations between gender norms and risk of adolescent HIV

**DOI:** 10.1016/j.eclinm.2022.101513

**Published:** 2022-06-26

**Authors:** Ribhav Gupta, Safa Abdalla, Valerie Meausoone, Nikitha Vicas, Iván Mejía-Guevara, Ann M. Weber, Beniamino Cislaghi, Gary L. Darmstadt

**Affiliations:** a Department of Epidemiology and Population Health, Stanford University School of Medicine, Stanford, CA, USA; b Department of Medicine, University of Minnesota School of Medicine, Minneapolis, MN, USA; c Global Center for Gender Equality, Department of Pediatrics, Stanford University School of Medicine, Stanford, CA, USA; d Department of Neuroscience, University of Texas – Dallas, Dallas, TX, USA; e Department of Medicine - Primary Care and Population Health, Stanford University School of Medicine, Palo Alto, CA, USA; f Stanford Aging and Ethnogeriatrics (SAGE) Research Center, Stanford University School of Medicine, Stanford, CA, USA; g School of Public Health, University of Nevada, Reno, NV, USA; h Department of Global Health and Development, London School of Hygiene and Tropical Medicine, London, UK; i Provider Network Data Science, Health Care Service Corporation (HCSC), Richardson, TX, USA

**Keywords:** Gender, Bias, Gender norms, Demographic and health surveys, HIV, Global health, Gender data, Data quality, SDG, Sustainable Development Goal, DHS, Demographic and Health Survey, PR, Prevalence ratio, RR, Relative risk, CI, Confidence interval, OR, Odds ratio, IPV, Intimate partner violence

## Abstract

**Background:**

Despite strides towards gender equality, inequalities persist or remain unstudied, due potentially to data gaps. Although mapped, the effects of key data gaps remain unknown. This study provides a framework to measure effects of gender- and age-imbalanced and missing covariate data on gender-health research. The framework is demonstrated using a previously studied pathway for effects of pre-marital sex norms among adults on adolescent HIV risk.

**Methods:**

After identifying gender-age-imbalanced Demographic and Health Survey (DHS) datasets, we resampled responses and restricted covariate data from a relatively complete, balanced dataset derived from the 2007 Zambian DHS to replicate imbalanced gender-age sampling and covariate missingness. Differences in model outcomes due to sampling were measured using tests for interaction. Missing covariate effects were measured by comparing fully-adjusted and reduced model fitness.

**Findings:**

We simulated data from 25 DHS surveys across 20 countries from 2005-2014 on four sex-stratified models for pathways of adult attitude-behaviour discordance regarding pre-marital sex and adolescent risk of HIV. On average, across gender-age-imbalanced surveys, males comprised 29.6% of responses compared to 45.3% in the gender-balanced dataset. Gender-age-imbalanced sampling significantly affected regression coefficients in 40% of model-scenarios (*N* = 40 of 100) and biased relative-risk estimates away from gender-age-balanced sampling outcomes in 46% (*N* = 46) of model-scenarios. Model fitness was robust to covariate removal with minor effects on male HIV models. No consistent trends were observed between sampling distribution and risk of biased outcomes.

**Interpretation:**

Gender-health model outcomes may be affected by sampling gender-age-imbalanced data and less-so by missing covariates. Although occasionally attenuated, the effect magnitude of gender-age-imbalanced sampling is variable and may mask true associations, thus misinforming policy dialogue. We recommend future surveys improve balanced gender-age sampling to promote research reliability.

**Funding:**

Bill & Melinda Gates Foundation grant OPP1140262 to Stanford University.

**Research in context**

**Evidence before this study**

Review of MEDLINE databases for articles regarding missing and biased data in gender-health research (keywords include “missing data”, “data bias”, “survey data”, or “gender equity”) published through January 2021 documents the breadth and depth of data gaps found in survey datasets from the community- to international-level; current literature is based solely on qualitative analyses, while the quantitative effects of data gaps on research outcomes remain unknown. Quantitative studies may uniquely demonstrate the importance of high-quality data, identify domains with significant data gaps, predict the effects of data gaps on the results of hypothesis testing, and inform which sampling strategies to prioritise to improve gender-health research reliability. We found no quantitative approaches to investigate the magnitude and direction of effects of data gaps in common datasets on gender-health research.

**Added value of this study**

This study provides a novel approach to measuring effects of data gaps in gender-health research, including use of a case study of four pathways regarding the effects of adult discordance in attitudes and behaviours regarding pre-marital sex on adolescent HIV risk to demonstrate the potential effects of various existing data gaps. Our findings indicate gender- and age-imbalanced sampling in global datasets can affect statistical outcomes of gender-health research and may modify interpretation for policy applications, while covariate missingness, or not including data on covariates, may present a lesser risk, particularly for well-designed models. Our research provides nuance to the ongoing conversation regarding effects of data gaps, demonstrates the variability in effects of different forms of data missingness and bias, and highlights the general unpredictability of their effects on analytic results and policy guidance.

**Implications of all the available evidence**

Inadequate quality data present a potential threat to misinterpretation of findings, mis-formulation of policies and misallocation of resources. Our findings support the existing call for improved gender- and age-balanced sampling practices in large, global datasets, and suggest that prioritising robust survey sampling methodologies to include subgroups currently under-sampled over collecting data on more covariates may yield more reliable insights to inform program and policy design to address gender inequalities. Future improvements to data collection practices will likely lead to improvements in health outcomes for individuals of all gender identities.

Alt-text: Unlabelled box

## Introduction

Gender equality, a key objective across the Sustainable Development Goals (SDGs) and the focus of SDG 5, is paramount to the development of societies and the health of individuals globally.^1^, ^2^, ^3^ A crucial component to advancing equality is addressing restrictive gender norms, the system of rules embedded in social institutions that govern the behaviour, attitudes, and access to resources for women and men.^1^^,^^2^^,^^4^^,^^5^ A number of interventions, from women's economic empowerment to violence prevention amongst boys, have resulted in improvements to gender health equality, including in women's health, care seeking behaviour for children, and infant mortality.^1^^,^^6^, ^7^, ^8^, ^9^

Despite these efforts, gender inequalities persist and contribute to worsened health outcomes.^6^^,^^7^^,^^10^^,^^11^ Our reliance on global health datasets ill-equipped for research at the gender-health nexus may be a central factor impeding progress, with interventions derived from datasets built on gender bias and restrictive gender norms perpetuating a cycle of gender inequalities and inadequate data collection.^1^^,^^12^ Gender data gaps – the systematic missingness of data on sectoral outcomes for one gender – can arise at any stage of data collection, from questionnaire design to sample selection to surveillance.^12^ Data 2X, under the United Nations Foundation, conducted a qualitative assessment of available survey data and mapped six sectors – including human security and health – with major data gaps for women and girls, which may ultimately bias our understanding of the health of all genders.^1^^,^^13^, ^14^, ^15^ For example, as in various global health surveys, the choice to prioritise child and reproductive health data in women's modules and limit men's modules to personal health questions may perpetuate assumptions of strict gendered roles, thus, for example, providing incomplete pictures of women's work hazards and men's role in child care.^13^^,^^16^, ^17^, ^18^ Likely a remnant of its history as the World Fertility Survey, the now Demographic and Health Survey (DHS), a commonly referenced global health surveillance program, routinely under-samples men, children and adolescents (5-19 years-old), and post-menopausal women (defined by the survey as over 49 years-old).^18^ Such practices may potentially underpower and bias analyses of health-related behaviours and outcomes, for example, on the role of communal decision-making (e.g. social norms and local governance structures) and their effect on healthcare-seeking behaviours (e.g., childhood vaccination, pain management, perinatal care) from the perspective of those under-sampled.^16^^,^^19^, ^20^, ^21^

Gender scholars have theorised that incomplete datasets may result in less effective gender-health research.^13^^,^^14^^,^^18^^,^^22^^,^^23^ We recently documented ways in which gender-related data are suboptimal, including data missingness, imbalance and bias.^12^ Now we ask, whether and to what degree these gender data inadequacies matter? If survey data are biased and do not represent all, how do we know whether the conclusions drawn are accurate, where the effects will manifest, to whom the implications apply, and for whom bias and discrimination may be created or perpetuated? Currently, the lack of quantitative methdologies and analysis leaves unclear to what extent study outcomes based on gender-biased data are limited in their explanatory potential and reliability.^14^^,^^15^^,^^17^^,^^18^^,^^23^

In this paper, we aim to provide a novel framework and set of quantitative methodologies to be used in conjunction with current approaches to evaluate the quality of datasets used for gender-health research and to demonstrate how insights from its application may lead to improved gender-health research reliability for all. Previously, we provided strategies to operationalise gender norms measurement using existing data.^1^^,^^24^ In particular, prior analyses using Zambian DHS data demonstrated that as community-level discordance or incongruity between adults’ professed attitudes and apparent behaviours regarding pre-marital sex widened, the risk for HIV acquisition increased for adolescent girls in that community.^2^^,^^24^^,^^25^ This survey (2007 Zambia DHS) was uniquely balanced in asking normative questions on pre-marital sex of men about men and women and of women about men and women, providing powerful quantitative insights on the effect this gender norm. Here we extend this analysis to gain insights into the effects of gender- and age-imbalanced and missing covariate data on gender-health outcomes. We ask whether balanced, complete data truly matter when measuring gender norms’ effects? The case study on the association of discordance between adult pre-marital sex attitudes and behaviours with adolescent HIV acquisition risk was uniquely chosen as it presents a previously studied gender-health pathway for which datasets are available with a wide range of gender data quality globally, permitting us to model the implications of existing data gaps.^1^^,^^14^

## Methods

### Overview

Based on existing datasets, we simulated the impact of gender- and age-imbalanced (referred to as gender-age-imbalanced) sampling and covariate missingness on measurements of the effect of communal norms amongst adults regarding premarital sex (self-reported attitudes and data-derived measures of behaviours; Appendix, equations [2] and [3]) upon adolescents’ risk of HIV infection. Our prior research – which showed that adolescents in Zambian communities with increasing communal discordance between attitudes and behaviors regarding pre-marital sex are at increasing risk of HIV infection,^1^^,^^25^^,^^26^ – relied on data for multiple individual-level (e.g., education status) and pooled community-level (e.g., intimate partner violence exposure) covariates. We hypothesised that our analytic models are sensitive to gender-age sampling imbalance and data missingness, and defined gender-age-imbalance as gender-age distributions that meaningfully diverge from the distribution of the balanced Zambian dataset.

To measure the impact of gender-age sampling imbalance and covariate missingness, we compared model outputs and fit when using a gender-age-balanced DHS dataset to those when using gender-age-imbalanced and/or incomplete, covariate restricted datasets. For the study, only data on sex (male or female) were available and served as a proxy for gender, for which we had no measures.

### Data sources

We reviewed all available DHS datasets (*N* = 301) for inclusion based on data availability, national HIV prevalence, and gender sampling distribution. We derived our criteria for gender-balanced data from those utilised by Weber et al., as these were the minimum requirements to build the necessary proxy covariates to study the association of gender norms with HIV status.^1^

**Figure 1:**
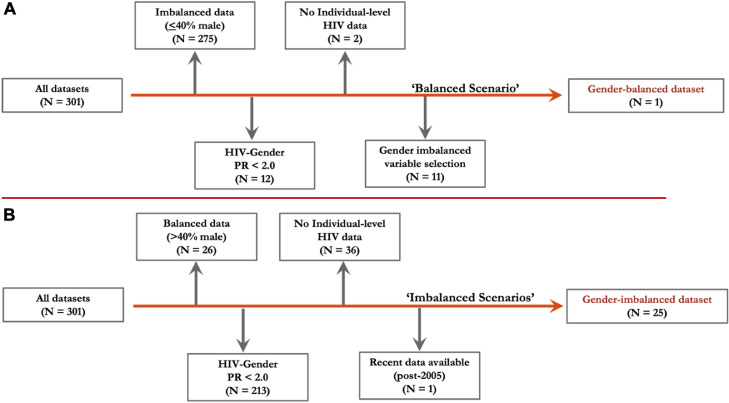
Inclusion and exclusion criteria for gender-balanced and gender-imbalanced datasets. The inclusion and exclusion criteria for identification of a baseline, gender-balanced Demographic and Health Survey (DHS) dataset (panel A) and series of gender-imbalanced DHS datasets (panel B) are provided. As the data informed specific sex-stratified pathways between adult pre-marital sex norms and adolescent risk of HIV, additional criteria unique to the case study were defined [e.g., HIV-gender prevalence ratio (PR) > 2.0].

The comparator DHS dataset – the 2007 Zambia DHS – was the only survey that included >40% male respondents across age groups, had a high gender HIV prevalence ratio (PR; PR ≥2.0) at the time of survey, as estimated using World Bank data (Figure 1, panel A),^27^ and had individual-level HIV data and responses for all relevant covariates (Figure 1). Datasets with gender-age imbalance and missing covariates were identified as the inverse of the comparator dataset. First, we excluded gender-balanced datasets (>40% of total respondents were males), which was an unusual occurrence (*N* = 26 excluded). Next, as the pathway is intended to explain gender disparities in HIV prevalence, we excluded surveys with a low gender HIV PR (women/men PR < 2.0) (*N* = 213 excluded). Third, we excluded surveys missing individual-level HIV data (*N* = 36 excluded). Finally, we standardised survey module availability to those in the gender-age-balanced dataset by selecting for surveys conducted post-2005 as this marked the transition to DHS-V for all countries (except Senegal; *N* = 1 excluded). This left 25 DHS datasets from which recent gender-imbalanced data with gender disparities in HIV prevalence were available (Figure 1, panel B). All datasets were confirmed to have a gender-age distribution dissimilar from the comparator distribution (>20% difference in proportion for at least one gender-age strata; Appendix, Table S1).

### Data simulation

To measure the effects of imbalanced sampling and missing covariate data on model results, subsets of the balanced, complete 2007 Zambia DHS dataset were randomly sampled to simulate the data gaps (i.e., gender-age sampling distributions and covariate missingness) found across the 25 gender-age-imbalanced and covariate-restricted datasets identified. First, we examined the effects of gender-age imbalance. We determined the distribution of eligible respondents by age group (15-19; 20-24; 25-49 years) and gender (male or female) for each imbalanced dataset. Eligible respondents included adolescents (15-24 years) who reported ever having sex and had received an HIV test, and all adults (25-49 years; individuals over 49 years are not commonly sampled). Next, we randomly sampled, with replacement, from all eligible respondents in the 2007 Zambia DHS dataset (*N* = 10,562) to match the gender- and age-unweighted distribution of gender-imbalanced datasets. Survey weights were recalculated to adjust for the probability of resampling (Appendix, equation [1]). Each simulated, gender-age-imbalanced dataset was appended to the original, balanced 2007 Zambia DHS copy after introducing a binary indicator to demarcate the balanced (2007 Zambian DHS) and the 25 imbalanced subsets (referred to as the imbalance term). This method was repeated to generate 100 unique datasets for each survey [gender-balanced (*N* = 1) and gender-imbalanced (*N* = 25)] to capture smooth distributions of responses (Appendix, Figures S1 and S2).

### Regression model

We assessed the effects of gender-age-imbalanced sampling on the previously published Poisson regression models designed by Weber et al.^1^ to study four pathways between communal discordance in adults’ (25-49 years) gender-specific attitudes and behaviours regarding pre-marital sex (referred to as the discordance term) and adolescents’ (15-24 years) individual risk of HIV. All models were sex-stratified, including same- and cross-gender pathways (adult female discordance ∼ adolescent female HIV; adult male discordance ∼ adolescent female HIV; adult female discordance ∼ adolescent male HIV; adult male discordance ∼ adolescent male HIV). Fully adjusted models incorporated individual-level demographic covariates (age, education, marital status, and urban/rural residence) and group-level covariates (e.g., intimate partner violence (IPV), alcohol use before sex) (Table 1). Recalculated DHS survey weights were applied to all regression models to adjust for the DHS multi-stage cluster sampling procedure and resampling probabilities.

**Table 1:** Patterns of data availability for model covariates across gender-imbalanced DHS surveys.

Covariates	Scenario 1 (*N* = 5)	Scenario 2 (*N* = 9)	Scenario 3 (*N* = 2)	Scenario 4 (*N* = 5)	Scenario 5 (*N* = 1)	Scenario 6 (*N* = 3)
**Individual-level**						
Age (15-19; 20-24)	✓	✓	✓	✓	✓	✓
Education (None; Primary; Secondary)	✓	✓	✓	✓	✓	✓
Marital status (Never/Formerly; Currently)	✓	✓	✓	✓	✓	✓
Residence (Urban; Rural)	✓	✓	✓	✓	✓	✓
**Group-level**						
History of intimate partner violence	✓	✓	Not available (Female HIV models)	Not available (Female HIV models)	✓	Not available (Female HIV models)
Belief: Married men only have sex with partner	✓	✓	✓	✓	✓	✓
Belief: Justified in beating women if refused sex	✓	✓	✓	✓	✓	✓
History of difference in sexual partners	✓	✓	✓	✓	Not available (Female HIV models)	Not available (Female HIV models)
History of alcohol use before sex	✓	Not available (Male HIV models)	✓	Not available (Male HIV models)	Not available (Male HIV models)	Not available (Male HIV models)

### Statistical analysis

#### Sampling imbalanced analysis

Statistical analyses tested the significance of independent and joint effects of data gaps (gender-age-imbalanced sampling and covariate missingness) on model pathways. In addition, an inter-survey analysis measured correlation between gender-age sampling (i.e., percent of adolescent respondents by gender) and risk of biased outcomes to provide a systematic interpretation of the effects of sampling imbalance.

The effects of gender-age-imbalanced sampling on model outcomes were measured using a test for interaction (Appendix, equations [2] and [3]). We first modified the four previously published same- and cross-gender regression models by including the imbalance indicator (binary term for gender- and age-balanced versus imbalanced sampling) as a covariate interacting with the explanatory variable, communal discordance (in pre-marital sex attitudes and behaviours). Next, the mean and standard deviation of regression beta-coefficients for discordance, imbalance, and interaction (of discordance and imbalance covariate) terms across the 100 bootstrapped datasets were estimated for each of the 25 imbalanced scenarios per model. The *p*-values for summary beta-coefficients were estimated with a statistical significance cut-off at *p* = 0.05. Statistical significance of the summary interaction coefficient term would indicate that the imbalanced datasets yield statistically different results compared to the balanced dataset. Additionally, the mean relative risk (RR) and 95% confidence interval (CI) of the discordance-HIV risk associations were calculated (Appendix, Tables S2 and S3).

#### Inter-survey pattern analyses

To better evaluate trends for how gender-age-imbalanced sampling affect model outcomes and to identify global predictors for significant changes to model outcomes, we performed an inter-survey analysis measuring the correlation between gender-age sample distributions and model outcomes across all imbalanced surveys. We built a logistic regression between gender-age sampling distributions of imbalanced surveys (*N* = 25) and whether the test for interaction was significant at the *p* = 0.05 level. This analysis was repeated for all four sex-stratified pathways. Finally, we estimated the odds ratio (OR) for how variation (i.e., increase or decrease) in the gender-age distribution affected the odds that regression outcomes differed from baseline (i.e., gender-balanced) outcomes.

To determine whether particular gender-age sampling distributions were correlated with the directionality and magnitude of changes to the model outcomes, a secondary inter-survey analysis was performed. A linear regression was utilised to compare the gender-age sample distributions and model outcomes (interaction coefficient and estimated PR) across imbalanced surveys (*N* = 25). This was repeated for all four sex-stratified models.

#### Missing covariate data

Statistical significance for the effect of missing covariate data on the sex-stratified models was measured using Wald tests. We generated a list of DHS questions used to build the covariates of each sex-stratified model and for each gender-age-imbalanced survey, flagging any covariates not asked or censured (Table 1). Using the balanced, 2007 Zambia DHS dataset, we performed a Wald test comparing the fit of the fully adjusted, sex-stratified models to those of reduced models where all identified patterns of flagged (missing) covariates were removed. Statistical significance of the F-statistic calculated would indicate that model fit for the balanced dataset was statistically different when a particular clustering of covariates was unavailable. In using the balanced dataset, for this analysis, we controlled for gender-age sampling.

We also evaluated joint effects of gender-age-imbalanced sampling and covariate missingness upon the discordance-HIV pathways using Wald tests. The test compared fit of fully adjusted and reduced variants of the interaction models (described above in test for interaction) for each scenario simulated. The test was applied to all 100 bootstrapped, merged datasets. Finally, the mean F-statistic and the *p*-value were estimated for each scenario to evaluate whether the covariate missingness scenario statistically changed model fitness.

All analyses were conducted using R programming language version 3.6.2 (R Foundation for Statistical Computing, Vienna, Austria) and Stata/SE software version 15.1 (StataCorp LP, College Station, TX, USA). Deidentified, publicly available data were used. The Stanford Institutional Review Board provided ethical approval through Protocol #40974; waiver of authorization, waiver of consent and waiver of HIPAA were granted. The analysis files are available online^28^ and all datasets utilised are available upon request from the DHS Program.

#### Role of the funding source

The funders of the study had no role in study design, data collection, data analysis, data interpretation, writing of the report, or the decision to submit the paper for publication. All authors had full access to all the data and approved the final paper for submission. The corresponding author had final responsibility to submit the paper for publication.

## Results

### Descriptive results

**Figure 2:**
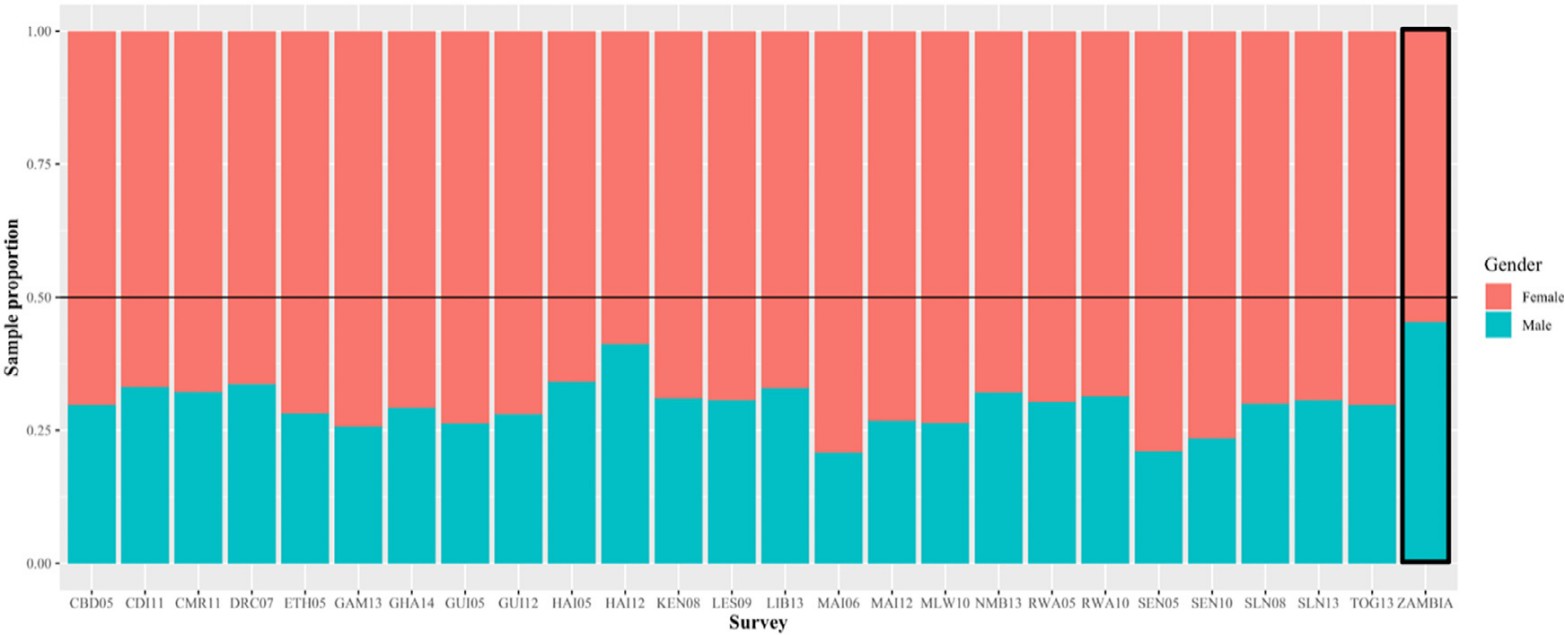
Unweighted gender distribution of total study-eligible respondents per survey sampled. We calculated the unweighted gender distribution of all respondents per Demographic and Health Survey (gender-balanced and imbalanced) included in the final sample. This included adolescents (15-24 years) with previous sexual experience and HIV testing data, and all adults (24-49 years). The female sample proportions are stacked on top of the male sample proportions. A horizontal line is added to indicate the global sex distribution. Note: Rightmost bar (ZAMBIA) is the gender distribution of the gender-balanced dataset. Key: CBD05: 2005 Cambodia; CDI11: 2011 Cote D'Ivore; CMR11: 2011 Cameroon; DRC07: 2007 Democratic Republic of the Congo; ETH05: 2005 Ethiopia; GAM13: 2013 Gambia; GHA14: 2014 Ghana; GUI05: 2005 Guinea; GUI12: 2012 Guinea; HAI05: 2005 Haiti; HAI12: 2012 Haiti; KEN08: 2008 Kenya; LES09: 2009 Lesotho; LIB13: 2013 Liberia; MAI06: 2006 Mali; MAI12: 2012 Mali; MLW10: 2010 Malawi; NMB13: 2013 Namibia; RWA05: 2005 Rwanda; RWA10: 2010 Rwanda; SEN05: 2005 Senegal; SEN10: 2010 Senegal; SLN08: 2008 Sierra Leone; SLN13: 2013 Sierra Leone; TOG13: 2013 Togo; ZAMBIA: 2007 Zambia.

In the gender-age-balanced, 2007 Zambia DHS dataset, 45.3% of total eligible respondents were male (Figure 2). Across all gender-age-imbalanced DHS datasets (*N* = 25; Figure 1), on average, male respondents comprised 29.6% of all eligible respondents, ranging from 20.8% (2006 Mali) to 41.2% (2012 Haiti) (Figure 2; Appendix, Table S1). Although the 2012 Haiti DHS survey initially met inclusion criteria for gender-imbalanced sampling (<40% of total respondents were male), after filtering for response eligibility (i.e., age, sexual history, available HIV data), >40% of included responses were male. Despite this apparent gender balance, the 2012 Haiti DHS dataset was retained as it met the apriori inclusion criteria, which relied on gender-imbalance at the total respondent level.

**Figure 3:**
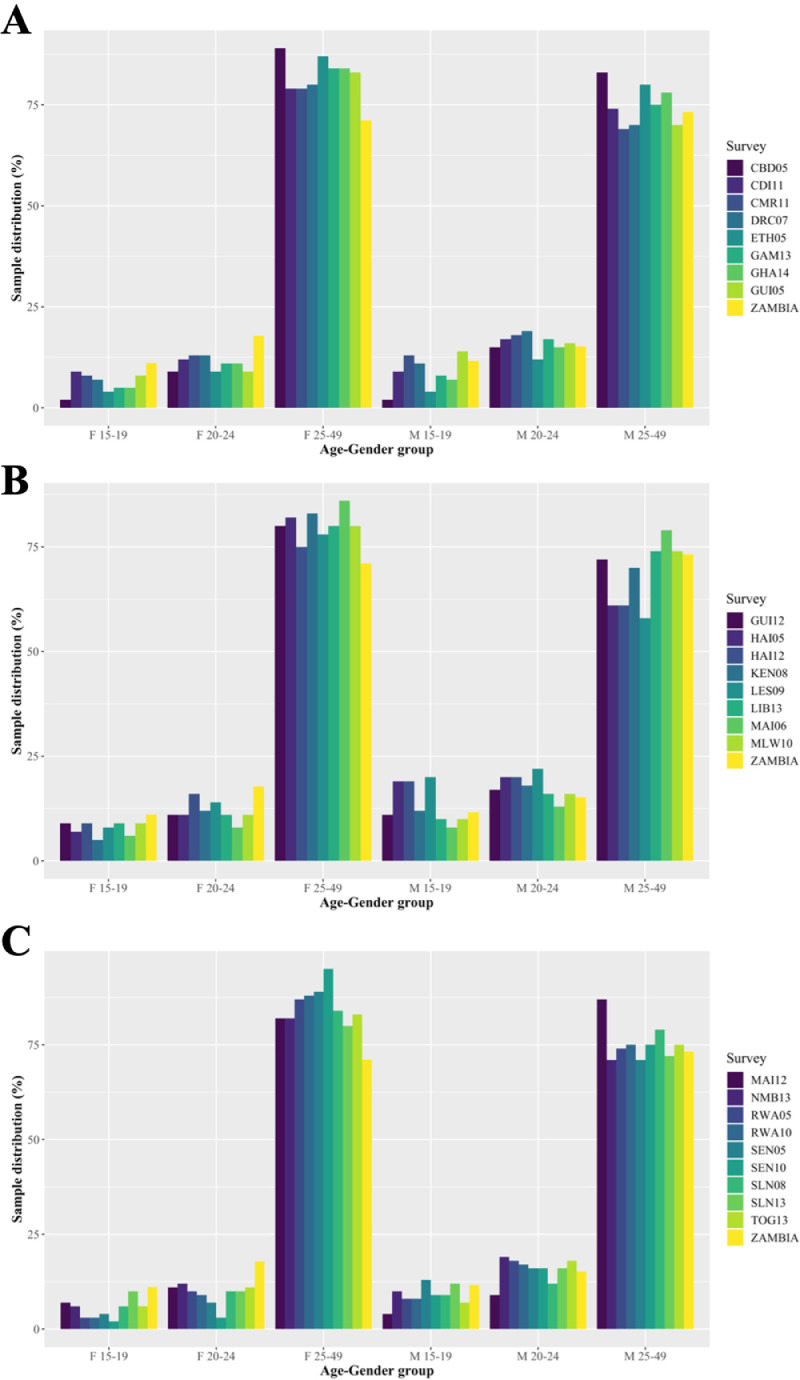
Unweighted gender-age distribution of study-eligible respondents per survey sampled. We calculated the unweighted gender-age distribution of all respondents per Demographic and Health Survey included in the final sample. Each panel (A-C) consists of the gender-age distribution from eight (panel C has nine) gender-imbalanced datasets, distributed in alphanumeric order, and from the gender-balanced DHS dataset (2007, Zambia; rightmost). Key: CBD05: 2005 Cambodia; CDI11: 2011 Cote D'Ivore; CMR11: 2011 Cameroon; DRC07: 2007 Democratic Republic of the Congo; ETH05: 2005 Ethiopia; GAM13: 2013 Gambia; GHA14: 2014 Ghana; GUI05: 2005 Guinea; GUI12: 2012 Guinea; HAI05: 2005 Haiti; HAI12: 2012 Haiti; KEN08: 2008 Kenya; LES09: 2009 Lesotho; LIB13: 2013 Liberia; MAI06: 2006 Mali; MAI12: 2012 Mali; MLW10: 2010 Malawi; NMB13: 2013 Namibia; RWA05: 2005 Rwanda; RWA10: 2010 Rwanda; SEN05: 2005 Senegal; SEN10: 2010 Senegal; SLN08: 2008 Sierra Leone; SLN13: 2013 Sierra Leone; TOG13: 2013 Togo; ZAMBIA: 2007 Zambia.

In the gender-age-balanced 2007 Zambia DHS dataset, 28.9% and 26.8% of eligible female and male respondents, respectively, were 15-24 years of age. Across all gender-imbalanced datasets, on average, 16.8% [range: 5.0% (2010 Senegal) to 25.0% (2012 Haiti)] of eligible female and 26.8% [range: 13.0% (2012 Mali) to 42.0% (2009 Lesotho)] of eligible male respondents were 15-24 years of age (Figure 3 and Appendix, Table S1).

Five patterns of missing covariates were identified across the gender-age-imbalanced surveys (Table 1) with 80% (*N* = 20 of 25) of imbalanced surveys missing all responses for at least one covariate. In a review of covariates missing from gender-age-imbalanced datasets that were required for female HIV risk models (adult female discordance ∼ adolescent female HIV; adult male discordance ∼ adolescent female HIV), seven surveys did not measure experiences of intimate partner violence, one survey did not measure the age difference of sexual partners, and three surveys did not include either. In a review of missing covariates required for male HIV risk models (adult female discordance ∼ adolescent male HIV; adult male discordance ∼ adolescent male HIV), 18 surveys did not measure alcohol use before sex.

### Effect of gender-age-imbalanced sampling

**Figure 4:**
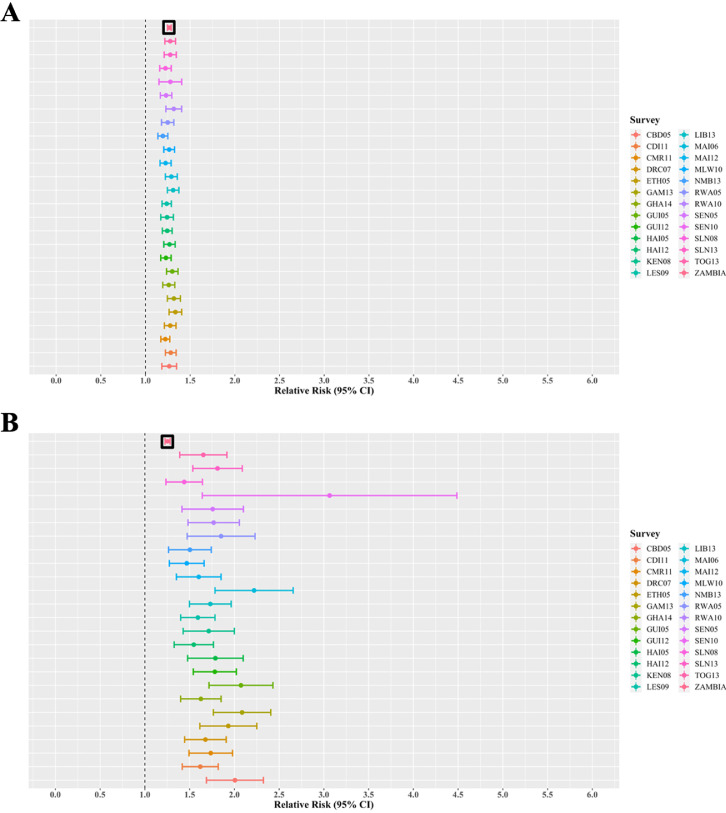
Relative risk estimations for the effect of pre-marital sex norms by adults on adolescent female HIV risk when using gender-imbalanced data. We estimated the mean and 95% confidence interval for the association (relative risk) between adolescent female risk of HIV and communal pre-marital sex norms amongst adult females (panel A) and adult men (panel B) using gender-imbalanced sampled datasets. The topmost bar represents the relative risk using the original, gender-balanced sampled dataset. The vertical line at the relative risk of one indicates no association. Key: CBD05: 2005 Cambodia; CDI11: 2011 Cote D'Ivore; CMR11: 2011 Cameroon; DRC07: 2007 Democratic Republic of the Congo; ETH05: 2005 Ethiopia; GAM13: 2013 Gambia; GHA14: 2014 Ghana; GUI05: 2005 Guinea; GUI12: 2012 Guinea; HAI05: 2005 Haiti; HAI12: 2012 Haiti; KEN08: 2008 Kenya; LES09: 2009 Lesotho; LIB13: 2013 Liberia; MAI06: 2006 Mali; MAI12: 2012 Mali; MLW10: 2010 Malawi; NMB13: 2013 Namibia; RWA05: 2005 Rwanda; RWA10: 2010 Rwanda; SEN05: 2005 Senegal; SEN10: 2010 Senegal; SLN08: 2008 Sierra Leone; SLN13: 2013 Sierra Leone; TOG13: 2013 Togo; ZAMBIA: 2007 Zambia.

Using simulated gender-age-imbalanced datasets, a 10% increase in discordance amongst female adults (regarding pre-marital sex attitudes and behaviors) was associated with a 19% to 33% increase in the risk of HIV infection amongst female adolescents [RR: 1.19 (2013 Namibia) to 1.33 (2005 Ethiopia)] (Figure 4). RRs calculated using imbalanced datasets did not differ from the baseline RR of 1.27 (95% CI: 1.25-1.29), calculated from gender-age balanced data, in all (*N* = 25) scenarios simulated (Figure 4). In testing for interaction, 8% (*N* = 2 of 25) of the gender-age-imbalanced scenarios yielded statistically significant differences in model outputs compared to gender-age-balanced model outcomes (Table 2).

**Table 2:** Interaction coefficient estimates of the test for interaction per sampled gender-imbalanced survey response distribution scenario for all pathways.

Survey	Adolescent female HIV ∼Adult female discordance (μ; 95% CI)	Adolescent female HIV ∼Adult male discordance (μ; 95% CI)	Adolescent male HIV ∼Adult female discordance (μ; 95% CI)	Adolescent male HIV ∼Adult male discordance (μ; 95% CI)
CBD05	0 (-0.02 – 0.01)	-0.02 (-0.04 – 0.01)	0.19 (0.16 – 0.23)*	0.2 (0.13 – 0.27)*
CDI11	0 (-0.01 – 0.02)	0 (-0.02 – 0.02)	0.06 (0.03 – 0.09)*	0.1 (0.05 – 0.14)*
CMR11	0.01 (-0.01 – 0.02)	-0.01 (-0.03 – 0.01)	0.02 (0 – 0.05)	0.04 (0 – 0.07)
DRC07	0 (-0.02 – 0.01)	0 (-0.02 – 0.02)	0.03 (0 – 0.05)*	0.07 (0.04 – 0.11)*
ETH05	-0.01 (-0.03 – 0)	-0.01 (-0.04 – 0.01)	0.15 (0.1 – 0.19)*	0.24 (0.17 – 0.31)*
GAM13	-0.01 (-0.03 – 0)	-0.03 (-0.05 – -0.01)*	0.08 (0.04 – 0.11)*	0.09 (0.05 – 0.14)*
GHA14	0 (-0.01 – 0.02)	0 (-0.02 – 0.03)	0.05 (0.02 – 0.08)*	0.1 (0.05 – 0.15)*
GUI05	0 (-0.02 – 0.01)	-0.02 (-0.04 – 0.01)	-0.04 (-0.08 – -0.01)*	0 (-0.06 – 0.06)
GUI12	0.01 (0 – 0.03)*	-0.01 (-0.03 – 0.01)	0.06 (0.03 – 0.1)*	0.08 (0.04 – 0.13)*
HAI05	0.01 (-0.01 – 0.02)	0 (-0.03 – 0.02)	-0.04 (-0.06 – -0.01)*	-0.04 (-0.07 – -0.01)*
HAI12	0 (-0.01 – 0.02)	0.01 (-0.01 – 0.03)	-0.05 (-0.08 – -0.03)*	-0.04 (-0.07 – -0.01)*
KEN08	0.01 (-0.01 – 0.02)	0 (-0.02 – 0.03)	0.03 (0 – 0.06)	0.04 (0 – 0.09)*
LES09	0.01 (-0.01 – 0.02)	-0.01 (-0.03 – 0.01)	-0.02 (-0.04 – 0.01)	0 (-0.03 – 0.03)
LIB13	0 (-0.02 – 0.01)	-0.01 (-0.03 – 0.02)	0.04 (0.01 – 0.07)*	0.07 (0.02 – 0.11)*
MAI06	0 (-0.02 – 0.01)	-0.04 (-0.06 – -0.01)*	0.05 (0 – 0.09)	0.03 (-0.03 – 0.09)
MAI12	0.02 (0 – 0.03)*	0.01 (-0.01 – 0.03)	0.1 (0.06 – 0.15)*	0.16 (0.11 – 0.22)*
MLW10	0.01 (0 – 0.02)	0.01 (-0.01 – 0.03)	0.04 (0.02 – 0.07)*	0.1 (0.05 – 0.15)*
NMB13	0.01 (0 – 0.03)	0.02 (-0.01 – 0.04)	0.05 (0.02 – 0.08)*	0.06 (0.02 – 0.1)*
RWA05	0 (-0.02 – 0.01)	-0.01 (-0.04 – 0.01)	0.1 (0.07 – 0.13)*	0.13 (0.1 – 0.17)*
RWA10	-0.01 (-0.03 – 0.01)	0 (-0.03 – 0.02)	0.09 (0.06 – 0.12)*	0.13 (0.08 – 0.18)*
SEN05	0.01 (-0.01 – 0.02)	0.01 (-0.02 – 0.03)	-0.01 (-0.05 – 0.02)	0.04 (-0.02 – 0.11)
SEN10	0.02 (0 – 0.05)	0.03 (-0.01 – 0.08)	0.05 (0.02 – 0.08)*	0.06 (0.02 – 0.1)*
SLN08	0.01 (0 – 0.03)	0.02 (0 – 0.05)	-0.01 (-0.04 – 0.03)	0.02 (-0.03 – 0.08)
SLN13	0.01 (0 – 0.03)	0 (-0.02 – 0.02)	0.01 (-0.02 – 0.04)	0.01 (-0.03 – 0.05)
TOG13	0 (-0.01 – 0.01)	0 (-0.02 – 0.03)	0.1 (0.07 – 0.13)*	0.16 (0.11 – 0.21)*

⁎ Indicates “imbalance” coefficient significant at *p* = 0.05 level (i.e. confidence interval does not include null). Key: CBD05: 2005 Cambodia; CDI11: 2011 Cote D'Ivore; CMR11: 2011 Cameroon; DRC07: 2007 Democratic Republic of the Congo; ETH05: 2005 Ethiopia; GAM13: 2013 Gambia; GHA14: 2014 Ghana; GUI05: 2005 Guinea; GUI12: 2012 Guinea; HAI05: 2005 Haiti; HAI12: 2012 Haiti; KEN08: 2008 Kenya; LES09: 2009 Lesotho; LIB13: 2013 Liberia; MAI06: 2006 Mali; MAI12: 2012 Mali; MLW10: 2010 Malawi; NMB13: 2013 Namibia; RWA05: 2005 Rwanda; RWA10: 2010 Rwanda; SEN05: 2005 Senegal; SEN10: 2010 Senegal; SLN08: 2008 Sierra Leone; SLN13: 2013 Sierra Leone; TOG13: 2013 Togo.

A 10% increase in discordance amongst male adults was associated with a 44% to 206% increase in the risk of HIV for female adolescents [RR: 1.44 (2008 Sierra Leone) to 3.06 (2010 Senegal)] (Figure 4). RRs calculated using imbalanced datasets differed from the baseline RR of 1.25 (95% CI: 1.22-1.28), calculated from balanced data, in 88% (*N* = 22) of scenarios simulated (Figure 4). In testing for interaction, 8% (*N* = 2 of 25) of the imbalanced scenarios yielded statistically significant differences in model outputs compared to balanced model outcomes (Table 2).

**Figure 5:**
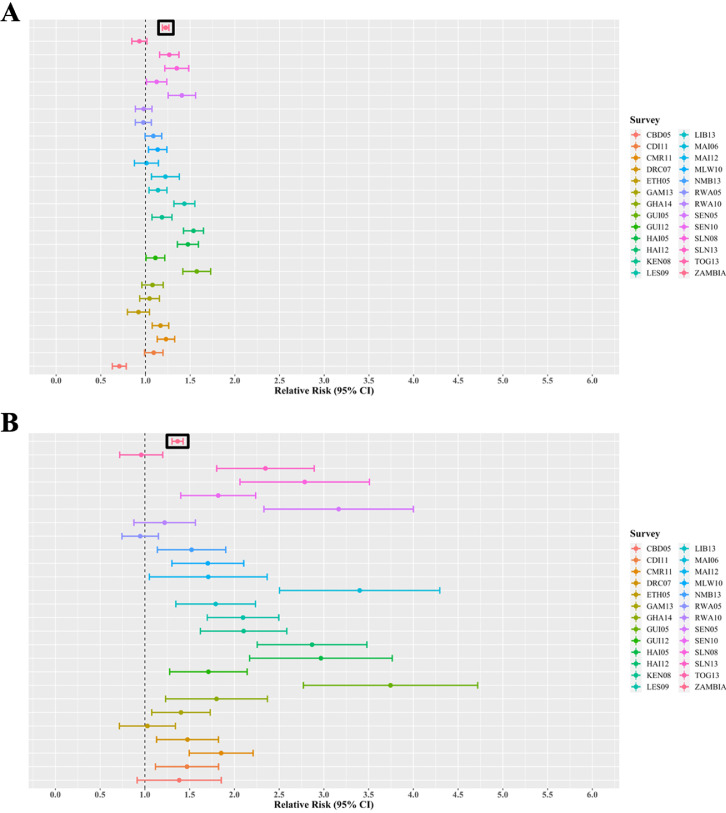
Relative risk estimations for the effect of pre-marital sex norms of adults on adolescent male HIV risk when using gender-imbalanced data. We estimated the mean and 95% confidence interval for the association (relative risk) between adolescent male risk of HIV and communal pre-marital sex norms amongst adult females (panel A) and adult men (panel B) using gender-imbalanced sampled datasets. The topmost bar represents the relative risk using the original, gender-balanced sampled dataset. The vertical line at the relative risk of one indicates no association. Key: CBD05: 2005 Cambodia; CDI11: 2011 Cote D'Ivore; CMR11: 2011 Cameroon; DRC07: 2007 Democratic Republic of the Congo; ETH05: 2005 Ethiopia; GAM13: 2013 Gambia; GHA14: 2014 Ghana; GUI05: 2005 Guinea; GUI12: 2012 Guinea; HAI05: 2005 Haiti; HAI12: 2012 Haiti; KEN08: 2008 Kenya; LES09: 2009 Lesotho; LIB13: 2013 Liberia; MAI06: 2006 Mali; MAI12: 2012 Mali; MLW10: 2010 Malawi; NMB13: 2013 Namibia; RWA05: 2005 Rwanda; RWA10: 2010 Rwanda; SEN05: 2005 Senegal; SEN10: 2010 Senegal; SLN08: 2008 Sierra Leone; SLN13: 2013 Sierra Leone; TOG13: 2013 Togo; ZAMBIA: 2007 Zambia.

A 10% increase in discordance amongst female adults was associated with a 29% decrease to 57% increase in the risk of HIV for male adolescents [RR: 0.71 (2005 Cambodia) to 1.57 (2005 Guinea)] (Figure 5). RRs calculated using imbalanced datasets differed from the baseline RR of 1.23 (95% CI: 1.19-1.26), calculated from balanced data, in 48% (*N* = 12) of scenarios simulated (Figure 5). In testing for interaction, 72% (*N* = 18 of 25) of the imbalanced scenarios yielded statistically significant differences in model outputs compared to balanced model outcomes (Table 2).

A 10% increase in discordance amongst male adults was associated with a 5% decrease to 374% increase in the risk of HIV for male adolescents [RR: 0.95 (2005 Rwanda) to 3.74 (2005 Guinea)] (Figure 5). RRs calculated using imbalanced datasets differed from the baseline RR of 1.37 (95% CI: 1.30-1.43), calculated from balanced data, in 48% (*N* = 12) of scenarios simulated (Figure 5). In testing for interaction, 72% (*N* = 18 of 25) of the imbalanced scenarios yielded statistically significant differences in model outputs compared to balanced model outcomes (Table 2).

### Inter-survey pattern analysis

An inter-survey analysis of the effect of gender-age-imbalanced sampling on the magnitude of change to model outcomes yielded variable results dependent on the outcome measured (Appendix, Figures S3 and S4). Adolescent (15-24 years) sample proportion was correlated with the magnitude and direction of changes to the estimated RR from baseline for three of the four sex-stratified models (Appendix, Tables S4 and S5 and Figure S4). Despite significant inter-survey correlations between sampling distributions (increased male and decreased female adolescent sampling) and changes to RR estimations, there was no consistent relation between adolescent sample proportion and odds model outcomes differed from those when using gender-balanced data (Appendix, Tables S6 and S7).

### Effect of missing covariate data

Using Wald tests, there were no statistically significant differences in the fit of fully adjusted models compared to the fit of reduced models from censuring most covariates (IPV and age difference between sexual partners; Appendix, Table S9). Exploratory sensitivity analysis demonstrated that removal of questions on IPV and age difference between partners had no meaningful effect on adolescent female HIV risk model effect size (Appendix, Table S8 and S9). When using gender-age-imbalanced datasets, there were no significant differences between fit of fully adjusted female or male HIV risk interaction and their reduced variants from censuring covariates (Appendix, Table S8 and S9).

## Discussion

We developed a framework to measure the effects of gender data gaps – specifically gender-age-imbalanced sampling and covariate missingness – on the reliability of gender health research outcomes. We applied this methodology to a previously published set of sex-stratified models evaluating the effect of communal discordance in adults’ attitudes and behaviours regarding pre-marital sex on the risk of HIV amongst adolescents (2). Broadly, imbalanced sampling on the basis of gender and age variably affected model results in an unpredictable manner; the most significant effects were seen on male adolescent HIV risk models, possibly due to under-sampling, whereas female adolescent HIV risk models were more robust to sampling variability. The fit of all models was generally robust to covariate missingness (i.e., covariates with no available data).

We simulated the gender (defined as a non-inclusive binary)-age sampling distribution of 25 DHS datasets across 20 countries and a 10-year timespan to capture different scenarios of data quality across geography and time. Model outcomes from gender-age-imbalanced data statistically differed from outcomes from balanced data in 40% of all model-scenarios (*N* = 40 of 100; Table 2). The measure of association, RR, was equally sensitive to differences in sampling distribution, with RRs from imbalanced data differing from estimates using balanced data in 46% (*N* = 46 of 100) of all model-scenarios (Table 2).

The majority (13 of 18) of model scenarios where imbalanced datasets produced both a statistically different association of the pathway and biased the association away from the baseline RR involved cross-gender models (i.e., the effect of female discordance on male HIV risk, or vice versa) (Table 2). In the cross-gender model for the effect of adult female discordance on adolescent male HIV risk, gender-age-imbalanced datasets occasionally biased the association to null (*N* = 7 of 25) and in one scenario produced a significant, protective effect (in relation to the baseline association which measured a significant increased risk) which may subsequently increase the risk of inaccurate conclusions (Figure 5 and Table 2). Of note, regarding the cross-gender model for the effect of adult male discordance on female adolescent HIV risk, the gender-age-imbalanced scenarios where model outputs differed statistically from balanced model outputs (i.e., significant test for interaction) were frequently distinct from the scenarios where the descriptive metrics differed quantitatively from one another (i.e., RR differed; Table 2 and Appendix, Table S2). This finding may be attributed to the strength of the baseline association, which may dampen the magnitude of imbalanced sampling effects. Overall, these findings indicate that although the statistical effects of imbalanced sampling may go unnoticed when drawing conclusions, in some cases, particularly for cross-gender models, imbalanced sampling can notably affect the reliability and interpretation of findings related to how gendered norms influence health outcomes. This is of note where gender-health datasets primarily collect same-gender data which can present a one-sided view on complex inter-gender dynamics.

Across all 25 imbalanced datasets, female adolescents (15-24 years) and male participants were routinely under-sampled compared to female adults (25-49 years; Appendix, Tables S1 and S10). On further examination, despite affects on model outcomes, we found no clear relationship between the degree of reduction or expansion in female and male adolescent participation, respectively, and the odds of biased results. Of note, as findings varied dependent on the proxy measure used for “reliability,” there may not be a single age group where increased sampling will significantly reduce risk of unreliable outcomes (Appendix, Tables S7 and S8 and Figures S3 and S4). However, we note that variance in association estimates was inversely correlated with gender-age balance as surveys with the lowest proportion of adolescent females sampled (i.e., Senegal 2010) had the greatest variance in RR estimates. While this is reflective of the smaller sample size from this subgroup, it is also indicative of a need to collectively improve sampling procedures (balance and inclusion) for all genders; evidence was less clear that this extends to all age groups.

This paper has some limitations posed by the methodology and the singular case utilised. First, given survey constraints, gender was defined as a cis-gender binary (female or male) derived from participant's stated sex and could not accurately capture a broader range of identities. Future surveys should consider inclusion of a distinct gender identity question to delineate critical differences.^12^ Additionally, the results presented are unique to the specific model evaluating the effect of adults’ communal discordance in pre-marital sex attitudes and behaviours on the risk of HIV in adolescents. Although the general lessons found here may be transferrable, a larger analysis of multiple gender health models across a spectrum of gender identities may provide further insights and validate the extent to which these findings are generalisable. However, in order to further test this approach to quantifying effects of gender data gaps, future analyses will require the increased availability of high-quality, adequately powered datasets necessary to provide the “true” outcomes for gender health models. As this framework is intended to compare research outcomes from variants of the same underlying dataset, it cannot directly compare outcomes across surveys given differences in the base population and responses. Finally, as this analysis can only be conducted following data collection, it cannot serve as a preventive strategy to improve research reliability or data quality.

Although the collection of high-quality datasets is critical to improving gender-health research reliability, future studies may also consider exploring the effectiveness of common data bias reduction practices to supplement these efforts. First, while not applicable to this study as DHS datasets require pre-calculated sample weighting to adjust for probability of inclusion, future analyses may explore the effectiveness of deriving survey weights for gender-age imbalanced datasets in improving research outcomes. In addition, while we only considered the effects of complete missing data (i.e., questions not asked at all), future studies utilising partially collected datasets may also explore effectiveness of common strategies such as imputation, expectation-maximisation, or substitution to reduce the effects of missing data on gender-health research.

We believe our study and framework provide novel and important nuance to the global conversation on how to better collect gender-health data and may inform future survey sampling methodologies to promote gender-health research reliability. While current health literature has primarily evaluated the effects of sub-ideal data quality from a theoretical lens or by relying on gender research across other sectors (e.g. agriculture and education), we begin to shed light on the disparate ways that sub-ideal data quality manifests in global health research.^13^, ^14^, ^15^^,^^18^^,^^23^ Our findings build upon years of such efforts from various sectors and approaches by demonstrating for the first time that gender data gaps can have a quantifiable effect on research reliability as had previously only been hypothesised.^1^^,^^10^^,^^29^, ^30^, ^31^ Optimistically, we find that these data gaps may not always affect the qualitative interpretation and policy implications of study outcomes. Moreover, the effects on analytic results and policy guidance were generally unpredictable, emphasising the importance of seeking to close data gaps and the futility of attempting to accomodate for them. Our findings support research exploring the effects of under-sampling subgroups (i.e., adolescent males and older females) by highlighting how the reliability of cross-gender data (data from men about women and vice-versa) plays a vital role in pathways concerned with gender norms. Our results also suggest that collection of high-quality gender-balanced samples, across age-groups, may be more pertinent than collection of sub-ideal data on more covariates, a hypothesis seldom found in existing literature.^30^ Although we recognise that, in practice, it is difficult for surveys to sufficiently sample individuals from all intersectional subgroups, our findings support efforts to prioritise gender balanced population representation in sample collection. Overall, our quantitative evaluation of the effects of gender data gaps on research reliability reinforces the global call for improved gender-stratified sampling practices across a breadth of health indicators.^10^^,^^15^^,^^29^, ^30^, ^31^

Our findings emphasise that if the data is not from all of us, it may not be for any of us. Data with built-in gender-age imbalance poses risks for deriving inaccurate conclusions, misinforming program and policy design, and recapitulating inequalities. As this systematic approach is retrospectively applied to new consortiums of global health datasets and domains of research as part of broader data review processes, predictions of the effect of gender data gaps are likely to improve. In the interim, it may remain difficult to gauge which gender-health analyses are at highest risk of bias. Moving forward, we believe that balanced sampling across and inclusion of all genders and ages can improve the reliability of gender-health research, including work on cross-gender normative influences.

## Funding

10.13039/100000865Bill & Melinda Gates Foundation grant OPP1140262 to Stanford University.

## Contributors

Conceptualization: V.M., G.L.D., and A.M.W; data curation: R.G. and N.V.; formal analysis: R.G. and N.V.; funding acquisition: G.L.D.; investigation: R.G., N.V., and V.M.; methodology: R.G., S.A., V.M., I.M.G., A.M.W, and G.L.D.; project administration: G.L.D.; resources: G.L.D.; supervision: G.L.D.; validation: S.A. and I.M.G.; visualization: R.G. and N.V.; writing – original draft: R.G.; writing – review and editing: all authors. All authors contributed intellectual content and approved the final draft. All authors had full access to the data in the study and take responsibility for the integrity and accuracy of the data analysis.

## Data sharing statement

DHS data are publicly available from: https://www.dhsprogram.com/Data/.

## Declaration of interests

The authors declare no conflicts of interests.

## Appendix Supplementary materials

Image, application 1
