# Supplementary material for: Effect of imbalanced sampling and missing data on associations between gender norms and risk of adolescent HIV
Source: eClinicalMedicine. 2022 Jun 26;50:101513. doi: 10.1016/j.eclinm.2022.101513 (PMC9241092; doi:10.1016/j.eclinm.2022.101513)
Supplement: Supplementary file 1 [file mmc1.docx]

**Supplemental Materials**

Gupta R, Abdalla S, Meausoone V, Vicas N, Mikhailov A, Mejía-Guevara I, Weber AM, Cislaghi B, Darmstadt GL. Effect of imbalanced sampling and missing data on associations between gender norms and risk of adolescent HIV.

**Contents**

Section 1: Technical appendix.……………..…………….……….……..………………….Page 3

Section 2: Supplemental figures and tables…………………..…………….………….....…Page 4

**Section 1: Technical appendix**

In this section, we provide additional methodologic details for sampling gender-age biased Demographic and Health Surveys (DHS) data and re-calculating relevant variables.

*Re-calculating survey weights*

In order to replicate the gender-age data imbalances present in gender-imbalanced datasets that met inclusion criteria (N=25), we repeatedly sampled at random with replacement from the gender-balanced 2007 Zambia DHS dataset to simulate data gaps present in imbalanced datasets. Each gender-imbalanced sampled dataset included the original number of eligible responses in the gender-balanced dataset (N=10,562) to maintain study power. This bootstrap process was repeated to generate 100 datasets per imbalanced scenario (N=25) and 100 datasets simulating the sample distribution of the original 2007 Zambia DHS dataset in order to better estimate model outcomes and account for sampling variation (See Figure S1 and Figure S2).

As a multi-stage cluster sample, DHS datasets include a sample weight variable that required re-calculation to account for the probability each given respondent was retained in simulated datasets given the new gender-age distribution.

Equation [1] was used to calculate the updated sample weights (${sweight}_{n}$) by multiplying the original weight (${sweight}_{i}$) by the probability each respondent would be included in the re-sampled dataset:

$$\left[ 1 \right] {sweight}_{imbalance}= \left( 1-\left( 1-\frac{1}{N_{a, g}} \right)^{p_{a,g}\sum N_{a,g}} \right){sweight}_{balance}$$

$$where,$$

$a$ = age cohort

$g$ = gender cohort

${sweight}_{i}$ = initial weights

$N_{a,g}$ = number of eligible respondents per gender-age cohort in the gender-balanced 2007 Zambia DHS dataset

$p_{a,g}$ = proportion of eligible respondents per gender-age cohort in the gender-imbalanced DHS dataset to be simulated

*Test for interaction analysis*

The effect of gender- and age-imbalanced sampling on the association of communal discordance in the attitudes and behavior regarding premarital sex on adolescent risk of HIV was measured using a test for interaction built on the base Poisson regression for the pathways. The four models were sex-stratified and included an interaction term of the discordance and imbalanced variables.

Equation [2] was used for the test for interaction on the association of both male and female adult discordance regarding premarital sex on female adolescent risk of HIV. Equation [3] was used for the test for interaction on the association of both male and female adult discordance regarding premarital sex on male adolescent risk of HIV.

$$\left[ 2 \right] \ln\left( Y_{i} \right)= \alpha+\beta_{1}d_{g,r}+\beta_{2}b_{i}+I_{1}d_{g,r}b_{i}+\beta_{3}a_{i}+\beta_{4}e_{i}+\beta_{5}m_{i}+\beta_{6}u_{i}+{\beta_{7}w_{r}+\beta_{8}j_{r}+\beta}_{9}v_{r}+\beta_{10}p_{r}$$

$$\left[ 3 \right] \ln\left( Y_{i} \right)= \alpha+\beta_{1}d_{g,r}+\beta_{2}b_{i}+I_{1}d_{g,r}b_{i}+\beta_{3}a_{i}+\beta_{4}e_{i}+\beta_{5}m_{i}+\beta_{6}u_{i}+\beta_{7}w_{r}+\beta_{8}l_{r}$$

$$where,$$

$Y$ = Binary outcome of whether adolescent tested positive for HIV.

$\alpha$ = Intercept.

$d$ = Communal adult discordance between attitudes towards and behaviours regarding premarital sex. Pre-marital sex attitudes are captured by a variable for *whether young men and women should until marriage for sexual intercourse.* Pre-marital sex behaviours are derived from two variables for whether (1*) the age at first sexual intercourse* is prior to (2) *the age at first marriage (or union).*

$b$ = Binary term for whether sample originated in gender-age balanced or imbalanced sampling dataset.

$a$ = Age of respondent.

$e$ = Highest level of education attained by respondent.

$m$ = Marital status of respondent.

$u$ = Whether respondent lives in a rural or urban location.

$w$ = Percent of married men who only have sex with their wives.

$j$ = Percent men who believe beating wives is justified if denied sex.

$v$ = Percent women who have experienced intimate partner violence.

$p$ = Percent women whose most recent sexual partner has an age difference greater than four years.

$l$ = Percent men who consumed alcohol prior to their last sex act.

$g$ = Gender of interest for discordance measure.

$i$ = Individual respondent.

$r$ = Aggregated at region.

All analyses were programmed in R version 3.6.2 (R Foundation for Statistical Computing; Vienna, Austria) and conducted in Stata/IC version 15.1 (StataCorp LP; College Station, United States), with the files and instructions for use available online (see main text reference to Github repository).

**Section 2: Supplemental Tables and Figures**

Table S1: Gender-age distribution of eligible respondent per survey.

Table S2: Relative risk estimates for the sex-stratified effect of pre-marital sex norms of adults on adolescent female HIV risk.

Table S3: Relative risk estimates for the sex-stratified effect of pre-marital sex norms of adults on adolescent male HIV risk.

Table S4: Linear association between gender-age sample distribution of imbalanced scenarios and value of the interaction coefficient.

Table S5: Linear association between gender-age sample distribution of imbalanced scenarios and difference in the relative risk from baseline.

Table S6: Odds ratio of the association between gender-age sample distribution of imbalanced scenarios and probability the interaction term is statistically significant.

Table S7: Odds ratio for the association between gender-age sample distribution of imbalanced scenarios and probability the difference in the relative risk from baseline is statistically significant.

Table S8: Relative risk of fully adjusted and models reduced of missing covariates using gender-balanced DHS data

Table S9: Wald test comparing fit of fully adjusted and reduced model variants from exclusion of covariate pairings missing in gender-imbalanced datasets

Table S10: Gender-stratified age distribution of all respondents per survey and of the national population for the corresponding country and year

Figure S1: Distribution of interaction coefficient estimates across resampled datasets per gender-imbalanced scenario for models of the effect of communal pre-marital sex norms on adolescent female HIV risk.

Figure S2: Distribution of interaction coefficient estimates across resampled datasets per gender-imbalanced scenario for models of the effect of communal pre-marital sex norms on adolescent male HIV risk.

Figure S3: Inter-survey regression of correlation between gender-age sampling distribution and value of the interaction coefficient across imbalanced scenarios.

Figure S4: Inter-survey regression of correlation between gender-age sampling distribution and difference in the relative risk estimate from baseline across imbalanced scenarios.

**Table S1. Gender-age distribution of eligible respondent per survey**

| **Survey** | **Gender-Age cohort** | **Percent of sample (%)** |
| --- | --- | --- |
| CBD05 | Female 15-19 | 1.41 (N=148) |
|  | Female 20-24 | 6.32 (N=668) |
|  | Female 25-49 | 62.48 (N=6599) |
|  | Male 15-19 | 0.60 (N=63) |
|  | Male 20-24 | 4.47 (N=472) |
|  | Male 25-49 | 24.73 (N=2612) |
| CDI11 | Female 15-19 | 6.02 (N=636) |
|  | Female 20-24 | 8.03 (N=848) |
|  | Female 25-49 | 52.85 (N=5657) |
|  | Male 15-19 | 2.98 (N=315) |
|  | Male 20-24 | 5.63 (N=595) |
|  | Male 25-49 | 24.49 (N=2587) |
| CMR11 | Female 15-19 | 5.42 (N=572) |
|  | Female 20-24 | 8.82 (N=931) |
|  | Female 25-49 | 53.56 (N=5657) |
|  | Male 15-19 | 4.19 (N=443) |
|  | Male 20-24 | 5.80 (N=613) |
|  | Male 25-49 | 22.21 (N=2347) |
| DRC07 | Female 15-19 | 4.64 (N=490) |
|  | Female 20-24 | 8.62 (N=910) |
|  | Female 25-49 | 53.04 (N=5602) |
|  | Male 15-19 | 3.71 (N=392) |
|  | Male 20-24 | 6.40 (N=676) |
|  | Male 25-49 | 23.59 (N=2492) |
| ETH05 | Female 15-19 | 2.88 (N=304) |
|  | Female 20-24 | 6.47 (N=683) |
|  | Female 25-49 | 62.55 (N=6607) |
|  | Male 15-19 | 1.13 (N=118) |
|  | Male 20-24 | 3.37 (N=356) |
|  | Male 25-49 | 23.60 (N=2493) |
| GAM13 | Female 15-19 | 3.71 (N=392) |
|  | Female 20-24 | 8.17 (N=863) |
|  | Female 25-49 | 62.42 (N=6592) |
|  | Male 15-19 | 2.06 (N=218) |
|  | Male 20-24 | 4.37 (N=462) |
|  | Male 25-49 | 19.27 (N=2035) |
| GHA14 | Female 15-19 | 3.54 (N=374) |
|  | Female 20-24 | 7.79 (N=823) |
|  | Female 25-49 | 59.47 (N=6281) |
|  | Male 15-19 | 2.04 (N=215) |
|  | Male 20-24 | 4.38 (N=463) |
|  | Male 25-49 | 22.78 (N=2406) |
| GUI05 | Female 15-19 | 5.90 (N=623) |
|  | Female 20-24 | 6.63 (N=700) |
|  | Female 25-49 | 61.17 (N=6461) |
|  | Male 15-19 | 3.68 (N=389) |
|  | Male 20-24 | 4.21 (N=445) |
|  | Male 25-49 | 18.41 (N=1944) |
| GUI12 | Female 15-19 | 6.48 (N=684) |
|  | Female 20-24 | 7.92 (N=837) |
|  | Female 25-49 | 57.60 (N=6084) |
|  | Male 15-19 | 3.08 (N=325) |
|  | Male 20-24 | 4.76 (N=503) |
|  | Male 25-49 | 20.16 (N=2129) |
| HAI05 | Female 15-19 | 4.61 (N=487) |
|  | Female 20-24 | 7.25 (N=766) |
|  | Female 25-49 | 54.04 (N=5708) |
|  | Male 15-19 | 6.48 (N=684) |
|  | Male 20-24 | 6.82 (N=720) |
|  | Male 25-49 | 20.80 (N=2197) |
| HAI12 | Female 15-19 | 5.29 (N=559) |
|  | Female 20-24 | 9.41 (N=994) |
|  | Female 25-49 | 44.10 (N=4658) |
|  | Male 15-19 | 7.83 (N=827) |
|  | Male 20-24 | 8.24 (N=870) |
|  | Male 25-49 | 25.13 (N=2654) |
| KEN08 | Female 15-19 | 3.45 (N=364) |
|  | Female 20-24 | 8.28 (N=875) |
|  | Female 25-49 | 57.27 (N=6049) |
|  | Male 15-19 | 3.72 (N=393) |
|  | Male 20-24 | 5.58 (N=589) |
|  | Male 25-49 | 21.70 (N=2292) |
| LES09 | Female 15-19 | 5.54 (N=585) |
|  | Female 20-24 | 9.71 (N=1025) |
|  | Female 25-49 | 54.05 (N=5709) |
|  | Male 15-19 | 6.14 (N=649) |
|  | Male 20-24 | 6.75 (N=713) |
|  | Male 25-49 | 17.81 (N=1025) |
| LIB13 | Female 15-19 | 6.04 (N=638) |
|  | Female 20-24 | 7.38 (N=779) |
|  | Female 25-49 | 53.68 (N=5670) |
|  | Male 15-19 | 3.29 (N=347) |
|  | Male 20-24 | 5.26 (N=556) |
|  | Male 25-49 | 24.35 (N=2572) |
| MAI06 | Female 15-19 | 4.75 (N=502) |
|  | Female 20-24 | 6.34 (N=670) |
|  | Female 25-49 | 68.11 (N=7194) |
|  | Male 15-19 | 1.66 (N=175) |
|  | Male 20-24 | 2.70 (N=285) |
|  | Male 25-49 | 16.44 (N=1735) |
| MAI12 | Female 15-19 | 5.12 (N=541) |
|  | Female 20-24 | 8.05 (N=850) |
|  | Female 25-49 | 60.03 (N=6339) |
|  | Male 15-19 | 1.07 (N=113) |
|  | Male 20-24 | 2.41 (N=255) |
|  | Male 25-49 | 23.32 (N=2463) |
| MLW10 | Female 15-19 | 6.62 (N=699) |
|  | Female 20-24 | 8.10 (N=856) |
|  | Female 25-49 | 58.88 (N=6219) |
|  | Male 15-19 | 2.64 (N=279) |
|  | Male 20-24 | 4.22 (N=446) |
|  | Male 25-49 | 19.54 (N=2064) |
| NMB13 | Female 15-19 | 4.07 (N=430) |
|  | Female 20-24 | 8.15 (N=861) |
|  | Female 25-49 | 55.68 (N=5881) |
|  | Male 15-19 | 3.21 (N=339) |
|  | Male 20-24 | 6.10 (N=644) |
|  | Male 25-49 | 22.79 (N=2407) |
| RWA05 | Female 15-19 | 2.09 (N=221) |
|  | Female 20-24 | 6.97 (N=736) |
|  | Female 25-49 | 60.64 (N=6405) |
|  | Male 15-19 | 2.42 (N=256) |
|  | Male 20-24 | 5.45 (N=576) |
|  | Male 25-49 | 22.43 (N=2368) |
| RWA10 | Female 15-19 | 2.06 (N=218) |
|  | Female 20-24 | 6.17 (N=652) |
|  | Female 25-49 | 60.37 (N=6376) |
|  | Male 15-19 | 2.51 (N=265) |
|  | Male 20-24 | 5.34 (N=564) |
|  | Male 25-49 | 23.55 (N=2487) |
| SEN05 | Female 15-19 | 3.16 (N=334) |
|  | Female 20-24 | 5.53 (N=584) |
|  | Female 25-49 | 70.31 (N=7426) |
|  | Male 15-19 | 2.73 (N=288) |
|  | Male 20-24 | 3.36 (N=355) |
|  | Male 25-49 | 14.91 (N=1575) |
| SEN10 | Female 15-19 | 1.53 (N=162) |
|  | Female 20-24 | 2.29 (N=242) |
|  | Female 25-49 | 72.68 (N=7675) |
|  | Male 15-19 | 2.11 (N=223) |
|  | Male 20-24 | 3.76 (N=397) |
|  | Male 25-49 | 17.63 (N=1861) |
| SLN08 | Female 15-19 | 4.20 (N=444) |
|  | Female 20-24 | 7.00 (N=739) |
|  | Female 25-49 | 58.80 (N=6210) |
|  | Male 15-19 | 2.70 (N=285) |
|  | Male 20-24 | 3.60 (N=380) |
|  | Male 25-49 | 23.70 (N=2503) |
| SLN13 | Female 15-19 | 6.93 (N=732) |
|  | Female 20-24 | 6.93 (N=732) |
|  | Female 25-49 | 55.44 (N=6856) |
|  | Male 15-19 | 3.68 (N=389) |
|  | Male 20-24 | 4.91 (N=519) |
|  | Male 25-49 | 22.11 (N=2334) |
| TOG13 | Female 15-19 | 4.21 (N=445) |
|  | Female 20-24 | 7.72 (N=815) |
|  | Female 25-49 | 58.27 (N=6154) |
|  | Male 15-19 | 2.09 (N=221) |
|  | Male 20-24 | 5.36 (N=566) |
|  | Male 25-49 | 22.35 (N=2361) |
| Reference  (ZAMBIA) | Female 15-19 | 6.03 (N=637) |
|  | Female 20-24 | 9.86 (N=1041) |
|  | Female 25-49 | 38.91 (N=4110) |
|  | Male 15-19 | 5.42 (N=572) |
|  | Male 20-24 | 6.78 (N=716) |
|  | Male 25-49 | 33.00 (N=3485) |

Key: CBD05: 2005 Cambodia; CDI11: 2011 Cote D’Ivore; CMR11: 2011 Cameroon; DRC07: 2007 Democratic Republic of the Congo; ETH05: 2005 Ethiopia; GAM13: 2013 Gambia; GHA14: 2014 Ghana; GUI05: 2005 Guinea; GUI12: 2012 Guinea; HAI05: 2005 Haiti; HAI12: 2012 Haiti; KEN08: 2008 Kenya; LES09: 2009 Lesotho; LIB13: 2013 Liberia; MAI06: 2006 Mali; MAI12: 2012 Mali; MLW10: 2010 Malawi; NMB13: 2013 Namibia; RWA05: 2005 Rwanda; RWA10: 2010 Rwanda; SEN05: 2005 Senegal; SEN10: 2010 Senegal; SLN08: 2008 Sierra Leone; SLN13: 2013 Sierra Leone; TOG13: 2013 Togo; ZAMBIA: 2007 Zambia.

**Table S2. Relative risk estimates for the sex-stratified effect of pre-marital sex norms of adults on adolescent female HIV risk**

| **Survey** | - **Adolescent female HIV ~**   **Adult female discordance**  **(RR; 95% CI)** | - **Adolescent female HIV ~**   **Adult male discordance**  **(RR; 95% CI)** |
| --- | --- | --- |
| CBD05 | 1.27 (1.18 – 1.35) | 2.01 (1.69 – 2.32)* |
| CDI11 | 1.28 (1.22 – 1.34) | 1.62 (1.42 – 1.82)* |
| CMR11 | 1.22 (1.17 – 1.27) | 1.74 (1.49 – 1.98)* |
| DRC07 | 1.28 (1.21 – 1.34) | 1.68 (1.44 – 1.91)* |
| ETH05 | 1.33 (1.26 – 1.41) | 1.93 (1.61 – 2.25)* |
| GAM13 | 1.32 (1.25 – 1.39) | 2.09 (1.76 – 2.41)* |
| GHA14 | 1.26 (1.19 – 1.32) | 1.63 (1.40 – 1.85)* |
| GUI05 | 1.30 (1.24 – 1.36) | 2.07 (1.72 – 2.43)* |
| GUI12 | 1.23 (1.17 – 1.29) | 1.78 (1.54 – 2.22)* |
| HAI05 | 1.27 (1.20 – 1.33) | 1.79 (1.48 – 2.10)* |
| HAI12 | 1.24 (1.19 – 1.30) | 1.55 (1.33 – 1.77)* |
| KEN08 | 1.24 (1.17 – 1.31) | 1.71 (1.43 – 2.00)* |
| LES09 | 1.24 (1.19 – 1.29) | 1.59 (1.40 – 1.78)* |
| LIB13 | 1.31 (1.25 – 1.37) | 1.73 (1.50 – 1.96)* |
| MAI06 | 1.29 (1.22 – 1.36) | 2.22 (1.78 – 2.66)* |
| MAI12 | 1.22 (1.16 – 1.29) | 1.60 (1.35 – 1.85)* |
| MLW10 | 1.27 (1.20 – 1.33) | 1.47 (1.27 – 1.66) |
| NMB13 | 1.19 (1.14 – 1.25) | 1.50 (1.26 – 1.74) |
| RWA05 | 1.25 (1.18 – 1.32) | 1.85 (1.47 – 2.23)* |
| RWA10 | 1.32 (1.23 – 1.40) | 1.77 (1.48 – 2.06)* |
| SEN05 | 1.23 (1.17 – 1.29) | 1.76 (1.41 – 2.10)* |
| SEN10 | 1.28 (1.15 – 1.41) | 3.06 (1.64 – 4.49)* |
| SLN08 | 1.22 (1.16 – 1.29) | 1.44 (1.24 – 1.64) |
| SLN13 | 1.28 (1.21 – 1.34) | 1.81 (1.54 – 2.09)* |
| TOG13 | 1.28 (1.22 – 1.34) | 1.65 (1.39 – 1.92)* |
| Reference (ZAMBIA) | 1.27 (1.25 – 1.29) | 1.25 (1.22 – 1.28) |

* “Imbalanced” RR significantly different from baseline (ZAMBIA) at p=0.05 level (i.e. confidence interval do not overlap).

Key: CBD05: 2005 Cambodia; CDI11: 2011 Cote D’Ivore; CMR11: 2011 Cameroon; DRC07: 2007 Democratic Republic of the Congo; ETH05: 2005 Ethiopia; GAM13: 2013 Gambia; GHA14: 2014 Ghana; GUI05: 2005 Guinea; GUI12: 2012 Guinea; HAI05: 2005 Haiti; HAI12: 2012 Haiti; KEN08: 2008 Kenya; LES09: 2009 Lesotho; LIB13: 2013 Liberia; MAI06: 2006 Mali; MAI12: 2012 Mali; MLW10: 2010 Malawi; NMB13: 2013 Namibia; RWA05: 2005 Rwanda; RWA10: 2010 Rwanda; SEN05: 2005 Senegal; SEN10: 2010 Senegal; SLN08: 2008 Sierra Leone; SLN13: 2013 Sierra Leone; TOG13: 2013 Togo; ZAMBIA: 2007 Zambia.

**Table S3. Relative risk estimates for the sex-stratified effect of pre-marital sex norms of adults on adolescent male HIV risk**

| **Survey** | - **Adolescent male HIV ~**   **Adult female discordance**  **(RR; 95% CI)** | - **Adolescent male HIV ~**   **Adult male discordance**  **(RR; 95% CI)** |
| --- | --- | --- |
| CBD05 | 0.71 (0.63 – 0.79) | 1.38 (0.91 – 1.85) |
| CDI11 | 1.09 (0.99 – 1.20) | 1.47 (1.11 – 1.82) |
| CMR11 | 1.23 (1.13 – 1.33) | 1.85 (1.50 – 2.21) |
| DRC07 | 1.17 (1.08 – 1.26) | 1.48 (1.13 – 1.82) |
| ETH05 | 0.92 (0.90 – 1.04) | 1.03 (0.72 – 1.34) |
| GAM13 | 1.05 (0.93 – 1.16) | 1.40 (1.08 – 1.73) |
| GHA14 | 1.08 (0.96 – 1.20) | 1.80 (1.23 – 2.37) |
| GUI05 | 1.57 (1.42 – 1.73) | 3.74 (2.77 – 4.72) |
| GUI12 | 1.11 (1.01 – 1.22) | 1.71 (1.28 – 2.14) |
| HAI05 | 1.47 (1.36 – 1.59 | 2.97 (2.17 – 3.76) |
| HAI12 | 1.54 (1.43 – 1.65) | 2.87 (2.26 – 3.48) |
| KEN08 | 1.18 (1.07 – 1.30) | 2.10 (1.62 – 2.59) |
| LES09 | 1.44 (1.32 – 1.55) | 2.10 (1.70 – 2.50) |
| LIB13 | 1.14 (1.04 – 1.24) | 1.79 (1.35 – 2.24) |
| MAI06 | 1.22 (1.07 – 1.38) | 3.40 (2.50 – 4.30) |
| MAI12 | 1.01 (0.88 – 1.14) | 1.71 (1.05 – 2.37) |
| MLW10 | 1.14 (1.03 – 1.24) | 1.70 (1.30 – 2.10) |
| NMB13 | 1.09 (0.99 – 1.18) | 1.52 (1.14 – 1.90) |
| RWA05 | 0.98 (0.89 – 1.07) | 0.95 (0.74 – 1.15) |
| RWA10 | 0.98 (0.89 – 1.07) | 1.22 (0.88 – 1.56) |
| SEN05 | 1.41 (1.25 – 1.56) | 3.17 (2.33 – 4.00) |
| SEN10 | 1.12 (1.01 – 1.24) | 1.82 (1.40 – 2.24) |
| SLN08 | 1.35 (1.22 – 1.48) | 2.79 (2.06 – 3.51) |
| SLN13 | 1.27 (1.16 – 1.37) | 2.35 (1.80 – 2.89) |
| TOG13 | 0.93 (0.85 – 1.02) | 0.96 (0.72 – 1.20) |
| Reference (ZAMBIA) | 1.23 (1.19 – 1.26) | 1.37 (1.30 – 1.43) |

* “Imbalanced” RR significantly different from baseline (ZAMBIA) at p=0.05 level (i.e. confidence interval do not overlap).

Key: CBD05: 2005 Cambodia; CDI11: 2011 Cote D’Ivore; CMR11: 2011 Cameroon; DRC07: 2007 Democratic Republic of the Congo; ETH05: 2005 Ethiopia; GAM13: 2013 Gambia; GHA14: 2014 Ghana; GUI05: 2005 Guinea; GUI12: 2012 Guinea; HAI05: 2005 Haiti; HAI12: 2012 Haiti; KEN08: 2008 Kenya; LES09: 2009 Lesotho; LIB13: 2013 Liberia; MAI06: 2006 Mali; MAI12: 2012 Mali; MLW10: 2010 Malawi; NMB13: 2013 Namibia; RWA05: 2005 Rwanda; RWA10: 2010 Rwanda; SEN05: 2005 Senegal; SEN10: 2010 Senegal; SLN08: 2008 Sierra Leone; SLN13: 2013 Sierra Leone; TOG13: 2013 Togo; ZAMBIA: 2007 Zambia.

**Table S4. Linear association between gender-age sample distribution of imbalanced scenarios and value of the interaction coefficient**

| **Model** | **Gender-Age cohort** | **Regression coefficient**  **(95% CI; p-value)** |
| --- | --- | --- |
| - *Adult female discordance ~*   *Adolescent female HIV risk* | Female 15-19 years | <0.01 (<0.01 – <0.01; p=0.038)* |
|  | Female 20-24 years | <0.01(<0.01 – <0.01; p=0.028)* |
|  | Female 25-49 years | NA |
| - *Adult male discordance ~*   *Adolescent female HIV risk* | Female 15-19 years | <0.01 (<0.01 – <0.01; p=0.693) |
|  | Female 20-24 years | <0.01 (<0.01 – <0.01; p=0.450) |
|  | Male 25-49 years | <0.01 (<0.01 – <0.01; p=0.787) |
| - *Adult female discordance ~*   *Adolescent male HIV risk* | Male 15-19 years | -0.01 (-0.02 – -0.01; p<0.001)* |
|  | Male 20-24 years | 0.01 (<0.01 – 0.02; p=0.004)* |
|  | Female 25-49 years | <0.01 (<0.01 – <0.01; p=0.499) |
| - *Adult male discordance ~*   *Adolescent male HIV risk* | Male 15-19 years | -0.04 (-0.06 – -0.03; p<0.001)* |
|  | Male 20-24 years | -0.02 (>-0.01 – -0.04; p=0.04)* |
|  | Female 25-49 years | -0.03 (-0.04 – -0.01; p=0.005)* |

* Indicates regression coefficient significant at p=0.05 level (i.e. confidence interval does not include null).

Note: “NA” denotes regression results could not be computed given model design.

**Table S5. Linear association between gender-age sample distribution of imbalanced scenarios and difference in the relative risk from baseline**

| **Model** | **Gender-Age cohort** | **Regression coefficient**  **(95% CI; p-value)** |
| --- | --- | --- |
| - *Adult female discordance ~*   *Adolescent female HIV risk* | Female 15-19 years | <0.01 (-0.01 – 0.01; p=0.970) |
|  | Female 20-24 years | <0.01(-0.01 – 0.01; p=0.503) |
|  | Female 25-49 years | NA |
| - *Adult male discordance ~*   *Adolescent female HIV risk* | Female 15-19 years | <0.01 (-0.05 – 0.05; p=0.953) |
|  | Female 20-24 years | -0.11 (-0.16 – -0.06; p<0.001)* |
|  | Male 25-49 years | -0.02 (-0.03 – <0.01; p=0.107) |
| - *Adult female discordance ~*   *Adolescent male HIV risk* | Male 15-19 years | 0.06 (0.05 – 0.07; p<0.001)* |
|  | Male 20-24 years | -0.04 (-0.06 – -0.03; p<0.001)* |
|  | Female 25-49 years | <0.01 (-0.01 – 0.01; p=0.834) |
| - *Adult male discordance ~*   *Adolescent male HIV risk* | Male 15-19 years | 0.37 (0.10 – 0.65; p=0.015)* |
|  | Male 20-24 years | -0.02 (-0.30 – 0.25; p=0.883) |
|  | Female 25-49 years | 0.19 (-0.09 – 0.46; p=0.194) |

* Indicates regression coefficient significant at p=0.05 level (i.e. confidence interval does not include null).

Note: “NA” denotes regression results could not be computed given model design.

**Table S6. Odds ratio of the association between gender-age sample distribution of imbalanced scenarios and probability the interaction term is statistically significant**

| **Model** | **Gender-Age cohort** | **Odds ratio**  **(95% CI; p-value)** |
| --- | --- | --- |
| - *Adult female discordance ~*   *Adolescent female HIV risk* | Female 15-19 years | 1.91 (0.72 – 5.00; p=0.190) |
|  | Female 20-24 years | 0.55 (0.22 – 1.40; p=0.212) |
|  | Female 25-49 years | NA |
| - *Adult male discordance ~*   *Adolescent female HIV risk* | Female 15-19 years | 1.20 (0.54 – 2.67; p=0.649) |
|  | Female 20-24 years | 1.13 (0.43 – 3.01; p=0.803) |
|  | Male 25-49 years | 1.08 (0.83 – 1.41; p=0.547) |
| - *Adult female discordance ~*   *Adolescent male HIV risk* | Male 15-19 years | 0.78 (0.55 – 1.12; p=0.177) |
|  | Male 20-24 years | 1.31 (0.79 – 2.15; p=0.294) |
|  | Female 25-49 years | 1.05 (0.81 – 1.37; p=0.708) |
| - *Adult male discordance ~*   *Adolescent male HIV risk* | Male 15-19 years | NA |
|  | Male 20-24 years | NA |
|  | Female 25-49 years | NA |

Note: “NA” denotes regression results could not be computed given model design.

**Table S7. Odds ratio for the association between gender-age sample distribution of imbalanced scenarios and probability the difference in the relative risk from baseline is statistically significant**

| **Model** | **Gender-Age cohort** | **Odds ratio**  **(95% CI; p-value)** |
| --- | --- | --- |
| - *Adult female discordance ~*   *Adolescent female HIV risk* | Female 15-19 years | NA |
|  | Female 20-24 years | NA |
|  | Female 25-49 years | NA |
| - *Adult male discordance ~*   *Adolescent female HIV risk* | Female 15-19 years | 0.97 (0.41 – 2.25; p=0.935) |
|  | Female 20-24 years | 0.40 (0.07 – 2.13; p=0.282) |
|  | Male 25-49 years | 0.69 (0.43 – 1.11; p=0.127) |
| - *Adult female discordance ~*   *Adolescent male HIV risk* | Male 15-19 years | 0.93 (0.69 – 1.25; p=0.629) |
|  | Male 20-24 years | 1.47 (0.89 – 2.43; p=0.129) |
|  | Female 25-49 years | 1.12 (0.88 – 1.43; p=0.350) |
| - *Adult male discordance ~*   *Adolescent male HIV risk* | Male 15-19 years | NA |
|  | Male 20-24 years | NA |
|  | Female 25-49 years | NA |

Note: “NA” denotes regression results could not be computed given model design.

**Table S8: Relative risk of fully adjusted and models reduced of missing covariates using gender-balanced DHS data**

| **Effect size (RR) of fully-adjusted and reduced models of adolescent female HIV risk** | | | | |
| --- | --- | --- | --- | --- |
| **Missing covariate(s) of reduced model** | **Female discordance ~**  **Female HIV risk**  (Fully-adjusted) | **Female discordance ~**  **Female HIV risk**  (Reduced) | **Male discordance ~**  **Female HIV risk**  (Fully-adjusted) | **Male discordance ~**  **Female HIV risk**  (Reduced) |
| History of interpersonal violence | 1.27  (CI: 1.11-1.45;  p <0.01) | 1.28  (CI: 1.12-1.47;  p <0.01) | 1.28  (CI: 1.05-1.56;  p = 0.02) | 1.29  (CI: 1.06-1.58;  p = 0.01) |
| History of age difference in sexual partners |  | 1.27  (CI: 1.11-1.46;  p <0.01) |  | 1.27  (CI: 1.04-1.54;  p = 0.02) |
| History of interpersonal violence & history of age difference in sexual partners |  | 1.28  (CI: 1.12-1.47;  p <0.01) |  | 1.27  (CI: 1.05-1.54;  p = 0.01) |
| **Effect size (RR) of fully-adjusted and reduced models of adolescent male HIV risk** | | | | |
|  | **Female discordance ~**  **Male HIV risk**  (Fully-adjusted) | **Female discordance ~**  **Male HIV risk**  (Reduced) | **Male discordance ~**  **Male HIV risk**  (Fully-adjusted) | **Male discordance ~**  **Male HIV risk**  (Reduced) |
| History of alcohol use during sex | 1.25  (CI: 0.90-1.73;  p = 0.18) | 1.20  (CI: 0.90-1.61;  p = 0.22) | 1.34  (CI: 0.93-1.92;  p = 0.12) | 1.23  (CI: 0.86-1.77;  p = 0.25) |

**Table S9: Wald test comparing fit of fully adjusted and reduced model variants from exclusion of covariate pairings missing in gender-imbalanced datasets**

| **F-statistics for Wald test of adolescent female HIV risk models** | | |
| --- | --- | --- |
| **Missing covariate(s) of reduced model** | **Adult female discordance ~**  **Adolescent female HIV risk** | **Adult male discordance ~**  **Adolescent female HIV risk** |
| History of interpersonal violence | 1.12 (p = 0.29) | 0.33 (p = 0.56) |
| History of age difference in sexual partners | 0.70 (p = 0.40) | 1.68 (p = 0.20) |
| History of interpersonal violence & history of age difference in sexual partners | 0.75 (p = 0.47) | 0.94 (p = 0.39) |
|  | | |
| **F-statistics for Wald test of adolescent male HIV risk models** | | |
|  | **Adult female discordance ~**  **Adolescent male HIV risk** | **Adult male discordance ~**  **Adolescent male HIV risk** |
| History of alcohol use during sex | 1.22 (p = 0.27) | 2.89 (p = 0.09) |

**Table S10. Gender-stratified age distribution of all respondents per survey and of the national population for the corresponding country and year**

| **Survey** | **Gender-Age cohort** | **Percent of all respondents per gender (%)** | **Percent of national population per gender (%)**^1–3^ |
| --- | --- | --- | --- |
| CBD05 | Female 15-19 | 21.67 | 5.90 |
|  | Female 20-24 | 17.95 | n/a |
|  | Female 25-49 | 60.38 | n/a |
|  | Male 15-19 | 25.40 | 6.13 |
|  | Male 20-24 | 17.56 | n/a |
|  | Male 25-49 | 57.03 | n/a |
| CDI11 | Female 15-19 | 19.85 | 5.50 |
|  | Female 20-24 | 19.75 | n/a |
|  | Female 25-49 | 60.40 | n/a |
|  | Male 15-19 | 17.99 | 5.47 |
|  | Male 20-24 | 17.27 | n/a |
|  | Male 25-49 | 54.74 | n/a |
| CMR11 | Female 15-19 | 23.27 | 5.39 |
|  | Female 20-24 | 20.21 | n/a |
|  | Female 25-49 | 50.74 | n/a |
|  | Male 15-19 | 22.42 | 5.44 |
|  | Male 20-24 | 16.56 | n/a |
|  | Male 25-49 | 50.74 | n/a |
| DRC07 | Female 15-19 | 20.85 | 5.15 |
|  | Female 20-24 | 22.29 | n/a |
|  | Female 25-49 | 56.86 | n/a |
|  | Male 15-19 | 19.36 | 5.19 |
|  | Male 20-24 | 17.91 | n/a |
|  | Male 25-49 | 53.75 | n/a |
| ETH05 | Female 15-19 | 23.11 | 5.22 |
|  | Female 20-24 | 18.60 | n/a |
|  | Female 25-49 | 58.29 | n/a |
|  | Male 15-19 | 21.18 | 5.28 |
|  | Male 20-24 | 17.22 | n/a |
|  | Male 25-49 | 52.30 | n/a |
| GAM13 | Female 15-19 | 24.07 | 5.58 |
|  | Female 20-24 | 20.53 | n/a |
|  | Female 25-49 | 55.40 | n/a |
|  | Male 15-19 | 22.69 | 5.58 |
|  | Male 20-24 | 20.65 | n/a |
|  | Male 25-49 | 48.84 | n/a |
| GHA14 | Female 15-19 | 18.69 | 5.13 |
|  | Female 20-24 | 16.72 | n/a |
|  | Female 25-49 | 64.59 | n/a |
|  | Male 15-19 | 20.26 | 5.36 |
|  | Male 20-24 | 14.13 | n/a |
|  | Male 25-49 | 53.46 | n/a |
| GUI05 | Female 15-19 | 20.52 | 5.63 |
|  | Female 20-24 | 14.28 | n/a |
|  | Female 25-49 | 65.20 | n/a |
|  | Male 15-19 | 21.42 | 5.65 |
|  | Male 20-24 | 13.99 | n/a |
|  | Male 25-49 | 49.12 | n/a |
| GUI12 | Female 15-19 | 21.82 | 5.72 |
|  | Female 20-24 | 17.65 | n/a |
|  | Female 25-49 | 60.53 | n/a |
|  | Male 15-19 | 21.07 | 5.80 |
|  | Male 20-24 | 15.07 | n/a |
|  | Male 25-49 | 51.67 | n/a |
| HAI05 | Female 15-19 | 25.48 | 5.63 |
|  | Female 20-24 | 18.52 | n/a |
|  | Female 25-49 | 56.00 | n/a |
|  | Male 15-19 | 24.99 | 5.66 |
|  | Male 20-24 | 17.12 | n/a |
|  | Male 25-49 | 46.35 | n/a |
| HAI12 | Female 15-19 | 24.32 | 5.27 |
|  | Female 20-24 | 19.58 | n/a |
|  | Female 25-49 | 56.10 | n/a |
|  | Male 15-19 | 23.39 | 5.31 |
|  | Male 20-24 | 17.00 | n/a |
|  | Male 25-49 | 47.85 | n/a |
| KEN08 | Female 15-19 | 20.93 | 5.47 |
|  | Female 20-24 | 20.65 | n/a |
|  | Female 25-49 | 58.42 | n/a |
|  | Male 15-19 | 22.02 | 5.45 |
|  | Male 20-24 | 17.89 | n/a |
|  | Male 25-49 | 54.05 | n/a |
| LES09 | Female 15-19 | 24.13 | 5.78 |
|  | Female 20-24 | 20.41 | n/a |
|  | Female 25-49 | 55.46 | n/a |
|  | Male 15-19 | 25.26 | 5.79 |
|  | Male 20-24 | 19.02 | n/a |
|  | Male 25-49 | 45.82 | n/a |
| LIB13 | Female 15-19 | 20.73 | 5.08 |
|  | Female 20-24 | 17.14 | n/a |
|  | Female 25-49 | 62.13 | n/a |
|  | Male 15-19 | 20.57 | 5.17 |
|  | Male 20-24 | 15.66 | n/a |
|  | Male 25-49 | 63.77 | n/a |
| MAI06 | Female 15-19 | 21.24 | 5.20 |
|  | Female 20-24 | 18.33 | n/a |
|  | Female 25-49 | 60.43 | n/a |
|  | Male 15-19 | 21.13 | 5.35 |
|  | Male 20-24 | 14.10 | n/a |
|  | Male 25-49 | 52.65 | n/a |
| MAI12 | Female 15-19 | 18.40 | 5.14 |
|  | Female 20-24 | 18.04 | n/a |
|  | Female 25-49 | 63.56 | n/a |
|  | Male 15-19 | 17.10 | 5.25 |
|  | Male 20-24 | 10.98 | n/a |
|  | Male 25-49 | 58.67 | n/a |
| MLW10 | Female 15-19 | 21.89 | 5.62 |
|  | Female 20-24 | 19.08 | n/a |
|  | Female 25-49 | 59.03 | n/a |
|  | Male 15-19 | 24.49 | 5.51 |
|  | Male 20-24 | 16.96 | n/a |
|  | Male 25-49 | 53.39 | n/a |
| NMB13 | Female 15-19 | 18.54 | 5.66 |
|  | Female 20-24 | 17.17 | n/a |
|  | Female 25-49 | 55.89 | n/a |
|  | Male 15-19 | 19.71 | 5.53 |
|  | Male 20-24 | 17.21 | n/a |
|  | Male 25-49 | 51.24 | n/a |
| RWA05 | Female 15-19 | 22.92 | 6.09 |
|  | Female 20-24 | 20.81 | n/a |
|  | Female 25-49 | 56.27 | n/a |
|  | Male 15-19 | 22.39 | 6.06 |
|  | Male 20-24 | 19.73 | n/a |
|  | Male 25-49 | 49.44 | n/a |
| RWA10 | Female 15-19 | 21.67 | 5.52 |
|  | Female 20-24 | 19.69 | n/a |
|  | Female 25-49 | 58.64 | n/a |
|  | Male 15-19 | 22.69 | 5.35 |
|  | Male 20-24 | 18.31 | n/a |
|  | Male 25-49 | 58.64 | n/a |
| SEN05 | Female 15-19 | 25.05 | 5.65 |
|  | Female 20-24 | 19.48 | n/a |
|  | Female 25-49 | 55.47 | n/a |
|  | Male 15-19 | 26.80 | 5.69 |
|  | Male 20-24 | 17.44 | n/a |
|  | Male 25-49 | 46.26 | n/a |
| SEN10 | Female 15-19 | 22.97 | 5.46 |
|  | Female 20-24 | 20.53 | n/a |
|  | Female 25-49 | 56.50 | n/a |
|  | Male 15-19 | 28.61 | 5.50 |
|  | Male 20-24 | 20.05 | n/a |
|  | Male 25-49 | 51.34 | n/a |
| SLN08 | Female 15-19 | 17.12 | 5.38 |
|  | Female 20-24 | 16.21 | n/a |
|  | Female 25-49 | 66.67 | n/a |
|  | Male 15-19 | 16.74 | 5.45 |
|  | Male 20-24 | 12.62 | n/a |
|  | Male 25-49 | 60.91 | n/a |
| SLN13 | Female 15-19 | 24.31 | 5.40 |
|  | Female 20-24 | 16.14 | n/a |
|  | Female 25-49 | 59.55 | n/a |
|  | Male 15-19 | 21.01 | 5.40 |
|  | Male 20-24 | 14.02 | n/a |
|  | Male 25-49 | 55.54 | n/a |
| TOG13 | Female 15-19 | 18.28 | 5.11 |
|  | Female 20-24 | 16.92 | n/a |
|  | Female 25-49 | 64.80 | n/a |
|  | Male 15-19 | 20.04 | 5.13 |
|  | Male 20-24 | 16.06 | n/a |
|  | Male 25-49 | 53.40 | n/a |
| Reference  (ZAMBIA) | Female 15-19 | 22.30 | 5.57 |
|  | Female 20-24 | 19.70 | n/a |
|  | Female 25-49 | 58.00 | n/a |
|  | Male 15-19 | 23.50 | 5.44 |
|  | Male 20-24 | 17.70 | n/a |
|  | Male 25-49 | 58.80 | n/a |

‘n/a’ references to data not available from the World Bank.

Key: CBD05: 2005 Cambodia; CDI11: 2011 Cote D’Ivore; CMR11: 2011 Cameroon; DRC07: 2007 Democratic Republic of the Congo; ETH05: 2005 Ethiopia; GAM13: 2013 Gambia; GHA14: 2014 Ghana; GUI05: 2005 Guinea; GUI12: 2012 Guinea; HAI05: 2005 Haiti; HAI12: 2012 Haiti; KEN08: 2008 Kenya; LES09: 2009 Lesotho; LIB13: 2013 Liberia; MAI06: 2006 Mali; MAI12: 2012 Mali; MLW10: 2010 Malawi; NMB13: 2013 Namibia; RWA05: 2005 Rwanda; RWA10: 2010 Rwanda; SEN05: 2005 Senegal; SEN10: 2010 Senegal; SLN08: 2008 Sierra Leone; SLN13: 2013 Sierra Leone; TOG13: 2013 Togo; ZAMBIA: 2007 Zambia.


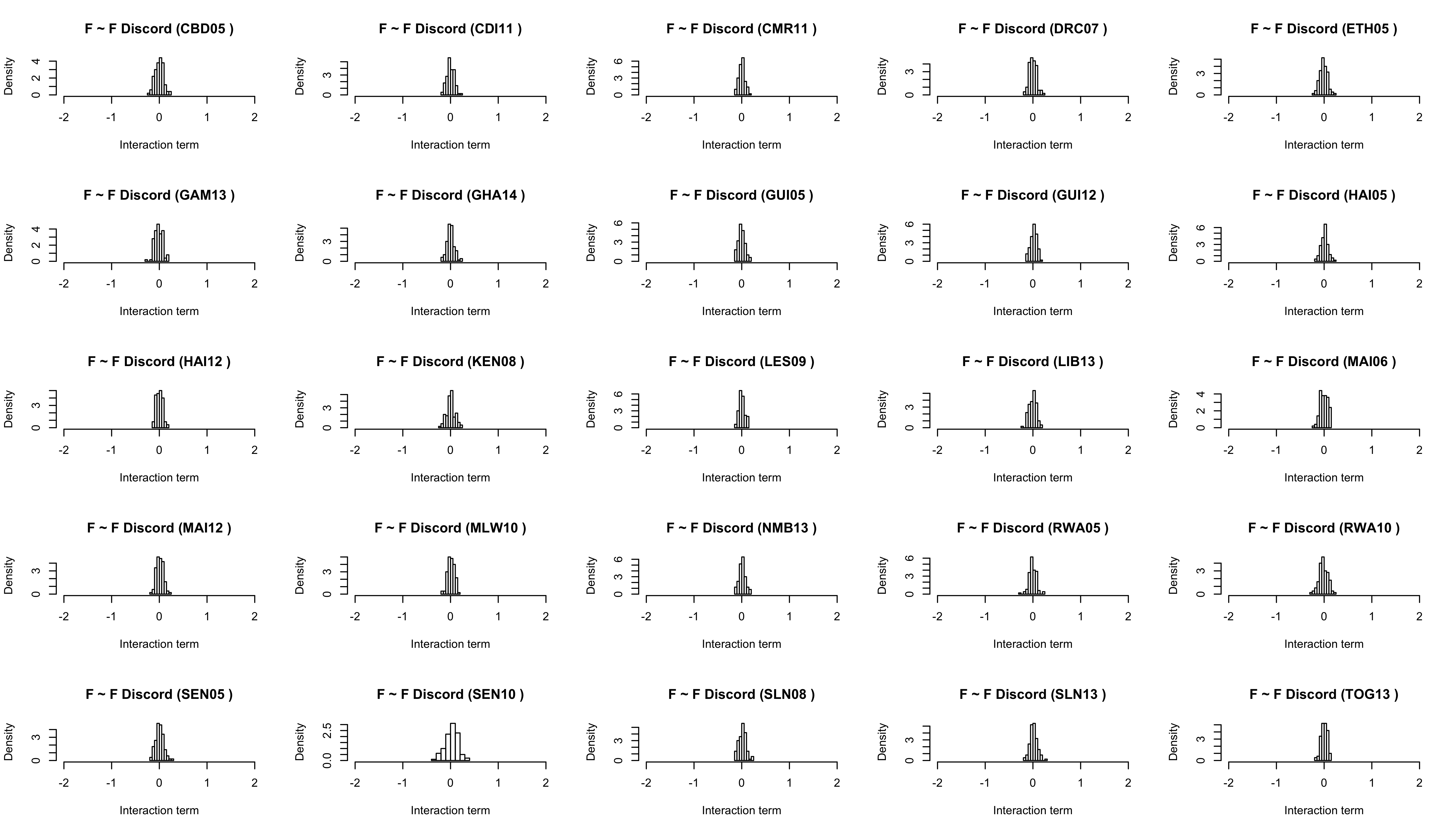

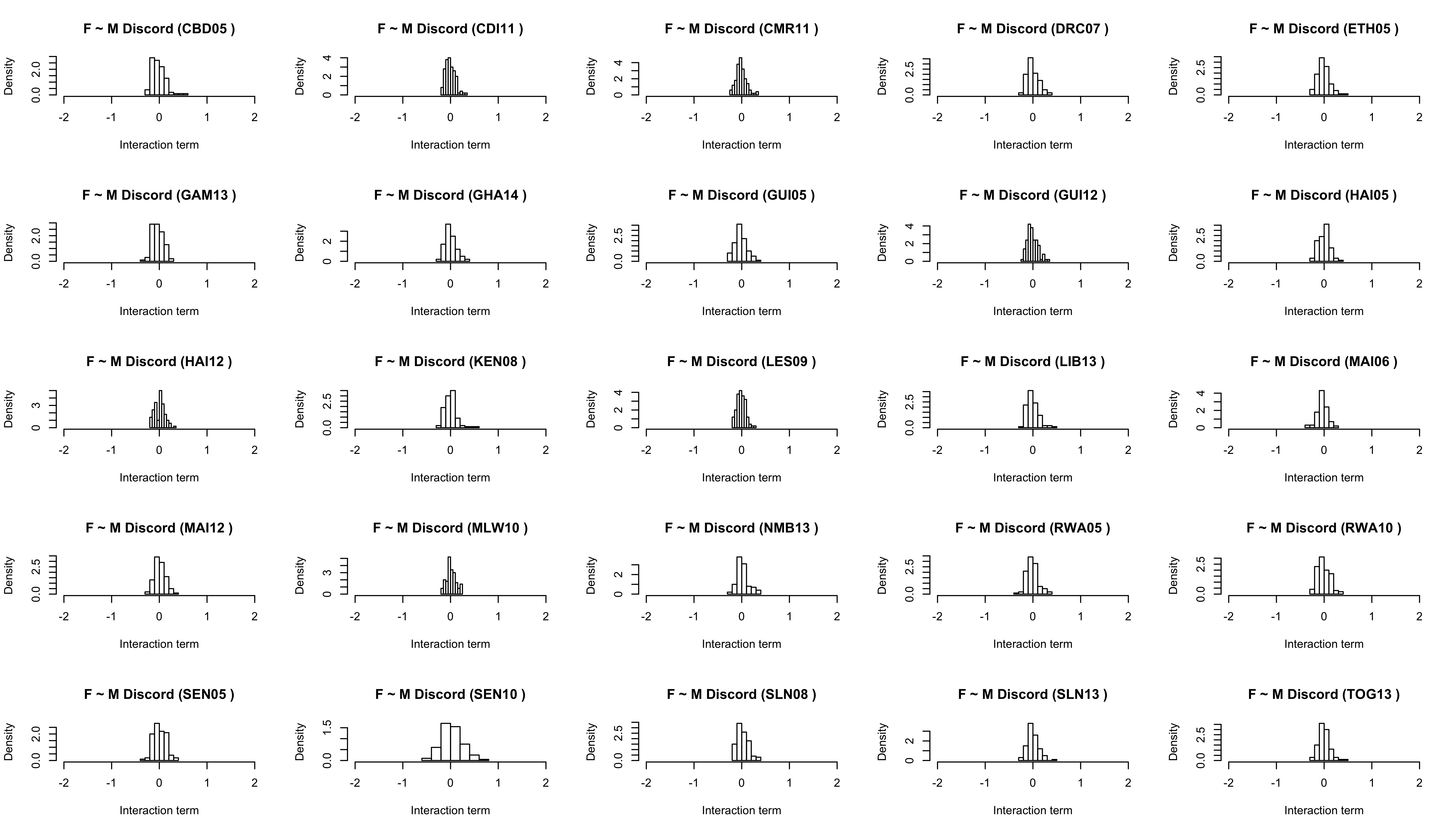


**A**

**B**

**Figure S1. Distribution of interaction coefficient estimates across resampled datasets per gender-imbalanced scenario for models of the effect of communal pre-marital sex norms on adolescent female HIV risk.** We estimated the interaction coefficient between bias and discordance covariates across 100 resampled datasets per gender-imbalanced scenario for models of the effect of communal pre-marital sex norms amongst adult females (panel A) and adult men (panel B) on adolescent female HIV risk. This was conducted to validate resampled datasets provided a smooth, gaussian distribution of model outcomes and to improve summary statistics.

Key: CBD05: 2005 Cambodia; CDI11: 2011 Cote D’Ivore; CMR11: 2011 Cameroon; DRC07: 2007 Democratic Republic of the Congo; ETH05: 2005 Ethiopia; GAM13: 2013 Gambia; GHA14: 2014 Ghana; GUI05: 2005 Guinea; GUI12: 2012 Guinea; HAI05: 2005 Haiti; HAI12: 2012 Haiti; KEN08: 2008 Kenya; LES09: 2009 Lesotho; LIB13: 2013 Liberia; MAI06: 2006 Mali; MAI12: 2012 Mali; MLW10: 2010 Malawi; NMB13: 2013 Namibia; RWA05: 2005 Rwanda; RWA10: 2010 Rwanda; SEN05: 2005 Senegal; SEN10: 2010 Senegal; SLN08: 2008 Sierra Leone; SLN13: 2013 Sierra Leone; TOG13: 2013 Togo.


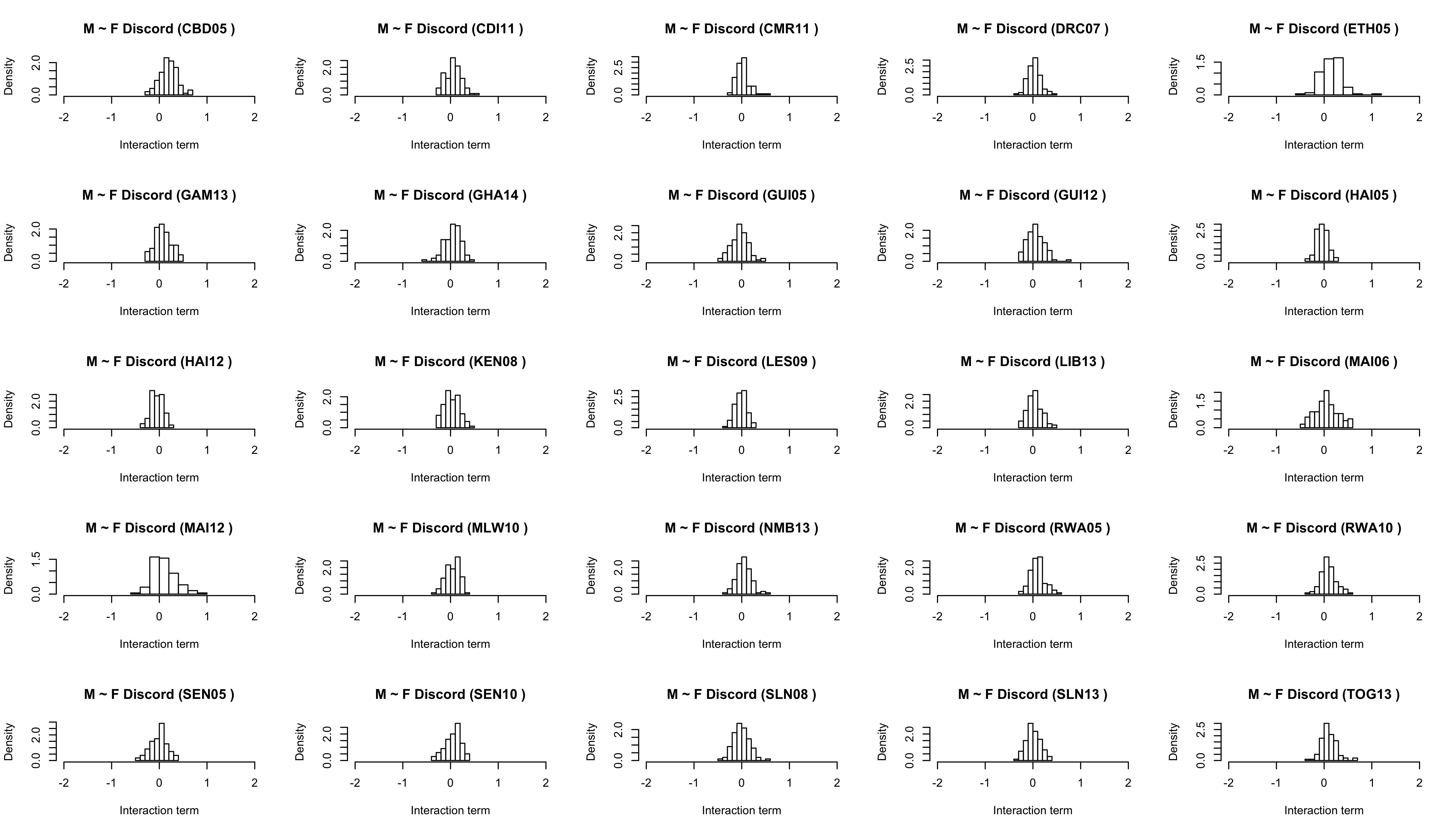

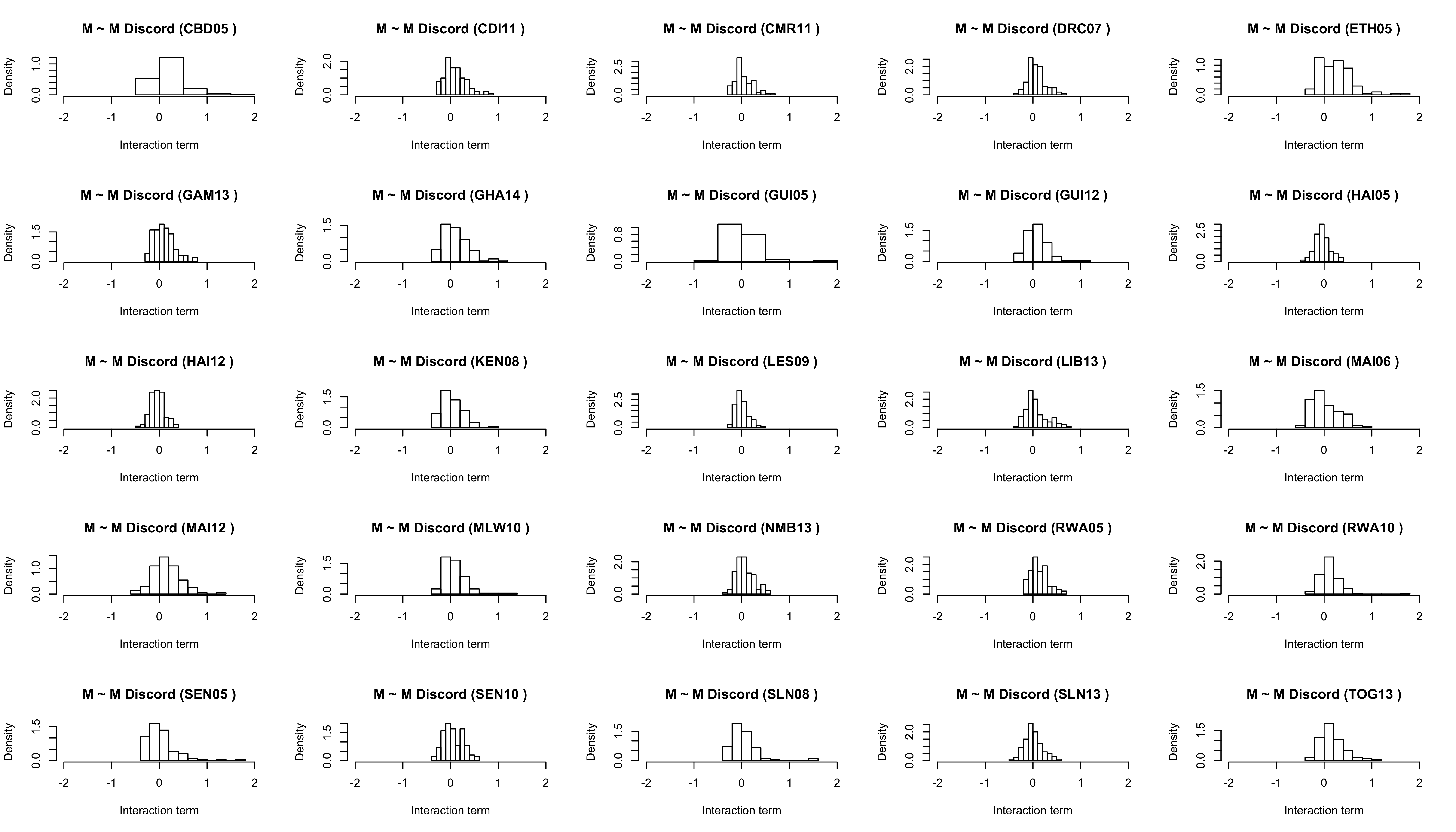


**A**

**B**

**Figure S2. Distribution of interaction coefficient estimates across resampled datasets per gender-imbalanced scenario for models of the effect of communal pre-marital sex norms on adolescent male HIV risk.** We estimated the interaction coefficient between bias and discordance covariates across 100 resampled datasets per gender-imbalanced scenario for models of the effect of communal pre-marital sex norms amongst adult females (panel A) and adult men (panel B) on adolescent male HIV risk. This was conducted to validate resampled datasets provided a smooth, gaussian distribution of model outcomes and to improve summary statistics. Note the large variation in interaction term estimates for GUI05 (panel B) is likely associated with the survey featuring the highest imbalance in gender-age sampling.

Key: CBD05: 2005 Cambodia; CDI11: 2011 Cote D’Ivore; CMR11: 2011 Cameroon; DRC07: 2007 Democratic Republic of the Congo; ETH05: 2005 Ethiopia; GAM13: 2013 Gambia; GHA14: 2014 Ghana; GUI05: 2005 Guinea; GUI12: 2012 Guinea; HAI05: 2005 Haiti; HAI12: 2012 Haiti; KEN08: 2008 Kenya; LES09: 2009 Lesotho; LIB13: 2013 Liberia; MAI06: 2006 Mali; MAI12: 2012 Mali; MLW10: 2010 Malawi; NMB13: 2013 Namibia; RWA05: 2005 Rwanda; RWA10: 2010 Rwanda; SEN05: 2005 Senegal; SEN10: 2010 Senegal; SLN08: 2008 Sierra Leone; SLN13: 2013 Sierra Leone; TOG13: 2013 Togo.

**
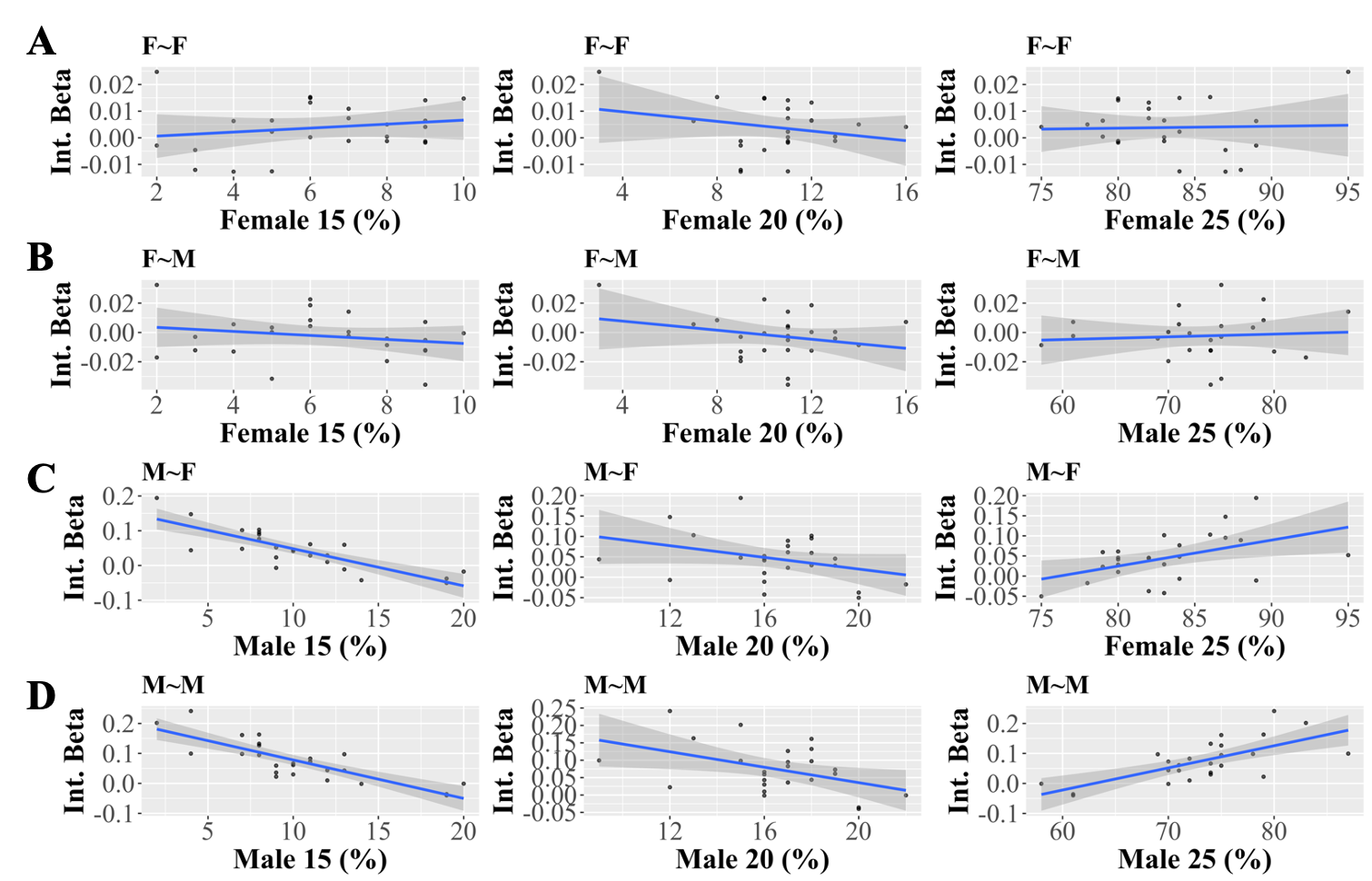
**

**Figure S3. Inter-survey regression of correlation between gender-age sampling distribution and value of the interaction coefficient across imbalanced scenarios.** We fit a linear regression to measure the association between gender-age sampling distribution and the interaction coefficient, a marker for the reliability of model outcomes for the gender-health pathway evaluated. The regression was fit using sample distributions and mean interaction coefficients for all gender-imbalanced scenarios simulated (N=25). This process was replicated for models of the effect of communal pre-marital sex norms amongst adult females (panel A) and adult men (panel B) on adolescent female HIV risk, and for models of the effect of communal pre-marital sex norms amongst adult females (panel C) and adult men (panel D) on adolescent male HIV risk.

**
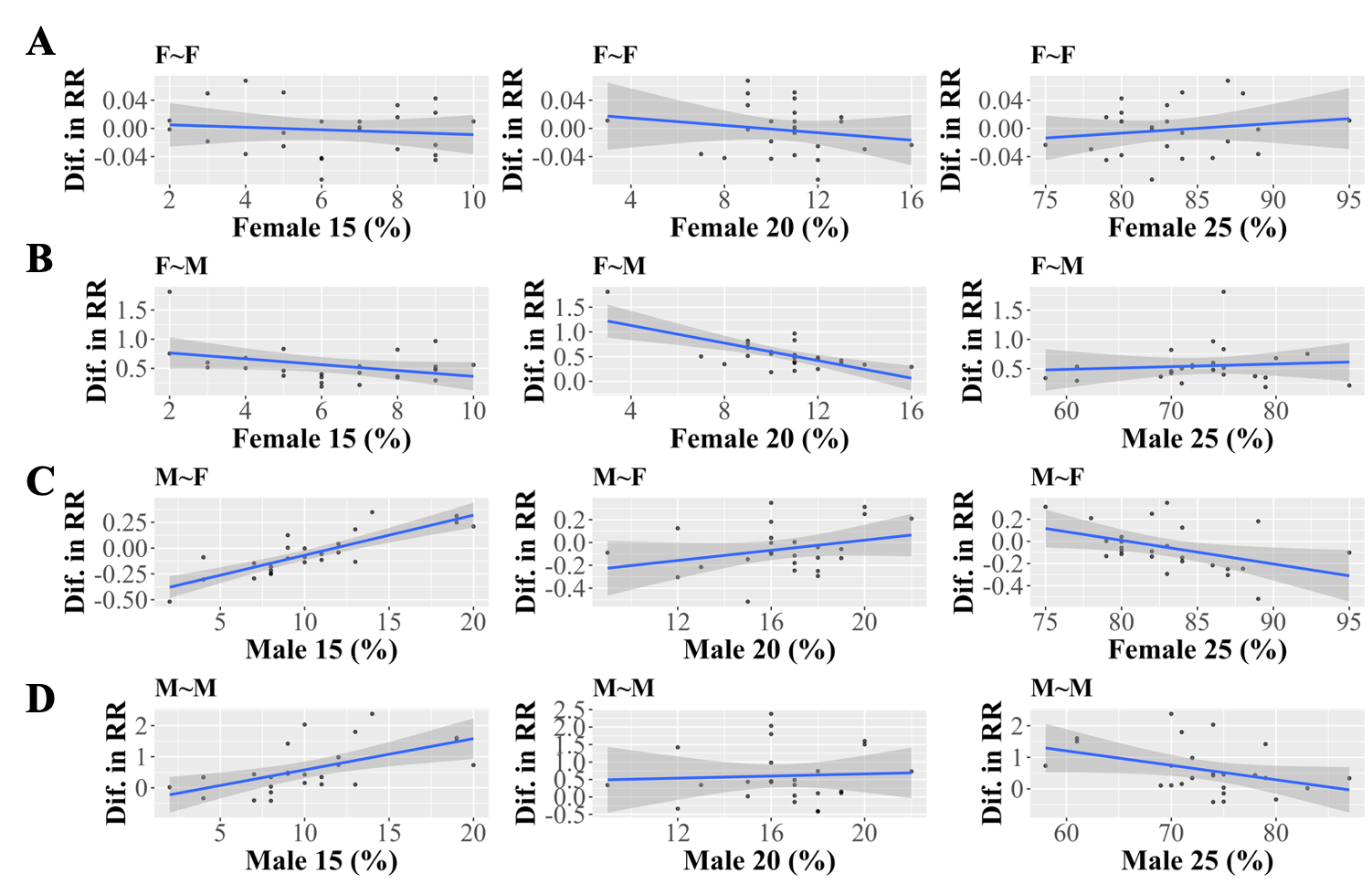
**

**Figure S4. Inter-survey regression of correlation between gender-age sampling distribution and difference in the relative risk estimate from baseline across imbalanced scenarios.** We fit a linear regression to measure the association between gender-age sampling distribution and the difference in the estimated relative risk from baseline (2007 Zambia DHS) for each gender-health pathway evaluated. The regression was fit using sample distributions and the mean change to the relative risk for all gender-imbalanced scenarios simulated (N=25). This process was replicated for models of the effect of communal pre-marital sex norms amongst adult females (panel A) and adult men (panel B) on adolescent female HIV risk, and for models of the effect of communal pre-marital sex norms amongst adult females (panel C) and adult men (panel D) on adolescent male HIV risk.

**References**

1. World Bank Group. Population, female (% of total population) [Internet]. 2019. Available from: https://data.worldbank.org/indicator/SP.POP.TOTL.FE.ZS

2. World Bank Group. Population ages 15-19, female (% of female population) [Internet]. World Bank Group; 2019. Available from: https://data.worldbank.org/indicator/SP.POP.1519.FE.5Y

3. World Bank Group. Population ages 15-19, male (% of male population) [Internet]. World Bank Group; 2019. Available from: https://data.worldbank.org/indicator/SP.POP.1519.MA.5Y
